# A 49-bp deletion of *PmAP2L* results in a double flower phenotype in *Prunus mume*

**DOI:** 10.1093/hr/uhad278

**Published:** 2023-12-19

**Authors:** Weichao Liu, Tangchun Zheng, Like Qiu, Xiaoyu Guo, Ping Li, Xue Yong, Lulu Li, Sagheer Ahmad, Jia Wang, Tangren Cheng, Qixiang Zhang

**Affiliations:** Beijing Key Laboratory of Ornamental Plants Germplasm Innovation & Molecular Breeding, National Engineering Research Center for Floriculture, Beijing Laboratory of Urban and Rural Ecological Environment, Engineering Research Center of Landscape Environment of Ministry of Education, Key Laboratory of Genetics and Breeding in Forest Trees and Ornamental Plants of Ministry of Education, School of Landscape Architecture, Beijing Forestry University, Beijing, China; Beijing Key Laboratory of Ornamental Plants Germplasm Innovation & Molecular Breeding, National Engineering Research Center for Floriculture, Beijing Laboratory of Urban and Rural Ecological Environment, Engineering Research Center of Landscape Environment of Ministry of Education, Key Laboratory of Genetics and Breeding in Forest Trees and Ornamental Plants of Ministry of Education, School of Landscape Architecture, Beijing Forestry University, Beijing, China; Beijing Key Laboratory of Ornamental Plants Germplasm Innovation & Molecular Breeding, National Engineering Research Center for Floriculture, Beijing Laboratory of Urban and Rural Ecological Environment, Engineering Research Center of Landscape Environment of Ministry of Education, Key Laboratory of Genetics and Breeding in Forest Trees and Ornamental Plants of Ministry of Education, School of Landscape Architecture, Beijing Forestry University, Beijing, China; Beijing Key Laboratory of Ornamental Plants Germplasm Innovation & Molecular Breeding, National Engineering Research Center for Floriculture, Beijing Laboratory of Urban and Rural Ecological Environment, Engineering Research Center of Landscape Environment of Ministry of Education, Key Laboratory of Genetics and Breeding in Forest Trees and Ornamental Plants of Ministry of Education, School of Landscape Architecture, Beijing Forestry University, Beijing, China; Beijing Key Laboratory of Ornamental Plants Germplasm Innovation & Molecular Breeding, National Engineering Research Center for Floriculture, Beijing Laboratory of Urban and Rural Ecological Environment, Engineering Research Center of Landscape Environment of Ministry of Education, Key Laboratory of Genetics and Breeding in Forest Trees and Ornamental Plants of Ministry of Education, School of Landscape Architecture, Beijing Forestry University, Beijing, China; Beijing Key Laboratory of Ornamental Plants Germplasm Innovation & Molecular Breeding, National Engineering Research Center for Floriculture, Beijing Laboratory of Urban and Rural Ecological Environment, Engineering Research Center of Landscape Environment of Ministry of Education, Key Laboratory of Genetics and Breeding in Forest Trees and Ornamental Plants of Ministry of Education, School of Landscape Architecture, Beijing Forestry University, Beijing, China; Beijing Key Laboratory of Ornamental Plants Germplasm Innovation & Molecular Breeding, National Engineering Research Center for Floriculture, Beijing Laboratory of Urban and Rural Ecological Environment, Engineering Research Center of Landscape Environment of Ministry of Education, Key Laboratory of Genetics and Breeding in Forest Trees and Ornamental Plants of Ministry of Education, School of Landscape Architecture, Beijing Forestry University, Beijing, China; Key Laboratory of National Forestry and Grassland Administration for Orchid Conservation and Utilization at College of Landscape Architecture, Fujian Agriculture and Forestry University, Fuzhou, China; Beijing Key Laboratory of Ornamental Plants Germplasm Innovation & Molecular Breeding, National Engineering Research Center for Floriculture, Beijing Laboratory of Urban and Rural Ecological Environment, Engineering Research Center of Landscape Environment of Ministry of Education, Key Laboratory of Genetics and Breeding in Forest Trees and Ornamental Plants of Ministry of Education, School of Landscape Architecture, Beijing Forestry University, Beijing, China; Beijing Key Laboratory of Ornamental Plants Germplasm Innovation & Molecular Breeding, National Engineering Research Center for Floriculture, Beijing Laboratory of Urban and Rural Ecological Environment, Engineering Research Center of Landscape Environment of Ministry of Education, Key Laboratory of Genetics and Breeding in Forest Trees and Ornamental Plants of Ministry of Education, School of Landscape Architecture, Beijing Forestry University, Beijing, China; Beijing Key Laboratory of Ornamental Plants Germplasm Innovation & Molecular Breeding, National Engineering Research Center for Floriculture, Beijing Laboratory of Urban and Rural Ecological Environment, Engineering Research Center of Landscape Environment of Ministry of Education, Key Laboratory of Genetics and Breeding in Forest Trees and Ornamental Plants of Ministry of Education, School of Landscape Architecture, Beijing Forestry University, Beijing, China

## Abstract

The double flower is an important trait with substantial ornamental value. While mutations in *PETALOSA* TOE-type or *AG* (*AGAMOUS*) genes play a crucial role in enhancing petal number in ornamental plants, the complete mechanism underlying the formation of double flowers remains to be fully elucidated. Through the application of bulked segregant analysis (BSA), we identified a novel gene, *APETALA2-like* (*PmAP2L*), characterized by a 49-bp deletion in double-flowered *Prunus mume*. β-Glucuronidase (GUS) staining and luciferase reporter assays confirmed that the 49-bp deletion in *PmAP2L* reduced its binding with *Pmu-miRNA172a*. Phylogenetic analysis and microsynteny analysis suggested that PmAP2L was not a *PETALOSA* TOE-type gene, and it might be a new gene controlling the formation of double flower in *P. mume*. Subsequently, overexpression of *PmAP2L-D* in tobacco led to a significant rise in the number of stamens and the conversion of stamens to petals. Furthermore, silencing of the homologue of *RC5G0530900* in rose significantly reduced the number of petals. Using transient gene expression in *P. mume* flower buds, we determined the functional differences between *PmAP2L-D* and *PmAP2-S* in controlling flower development. Meanwhile, DNA-affinity purification sequencing (DAP-seq), yeast hybrid assays and luciferase reporter assays indicated that PmAP2L negatively regulated the floral organ identity genes by forming a repressor complex with PmTPL and PmHDA6/19. Overall, these findings indicate that the variation in *PmAP2L* is associated with differences in the regulation of genes responsible for floral organ identity, providing new insights into the double-flower trait and double-flower breeding in plants.

## Introduction

The flowers of angiosperms undergo at least four developmental processes: floral organ initiation and determination, morphogenesis and maturation [1, 2]. Changes in these processes induce significant variations in flowering time, size, color, arrangement, and the number of floral organs, thereby contributing to the diverse and vibrant floral palette observed in angiosperms [1, 3]. The initiation and determination of floral organs are regulated by the ABC model of flower development, which was first inferred through a set of homeotic genes identified in *Arabidopsis thaliana* and *Antirrhinum majus* [4–6]. According to the ABCDE model, five classes of floral organ identity genes control the four types of floral organs. The identity of sepals is regulated by the combination of A and E class genes, whereas the determination of petals involves A, B, and E class genes. Carpels, on the other hand, are defined by the presence of B, C, and E class genes [6].

*AGAMOUS* (*AG*), as the earliest cloned C-class gene, serves a dual role in both terminating and maintaining the floral meristem [7, 8]. In *ag* mutants, A class genes exhibit persistent expression in the third and fourth whorl of flower organs, resulting in ‘flower-within-flower’ phenotype [4, 8]. Hence, numerous studies have concentrated on the genetic variations caused by *AG* gene, especially the double flower trait. Analysis of *ThtAG1* alleles in *Thalictrum thalictroides* showed that the insertion of a 2209 bp long terminal repeat (LTR) in exon 4 induced a nonsense mutation with K-domain deletions, and the mutated protein abolished interactions with the E class protein ThtSEP3 [9]. *AG* from the double flowers in *Prunus serrulate* and *Magnolia stellata* showed exon skipping as compared to the single flowers, whereas *AG* in Japanese Kerria (*Kerria japonica*) and yellowhorn (*Xanthoceras sorbifolium*) showed a transposon-mediated insertion [10–13]. Furthermore, *AP2*, a member of A-class genes, antagonizes the transcriptional activity of the C-class genes and is closely associated with the formation of double flowers. In strong *ap2* mutants, the expression of *AG* is intensified in the first and second floral whorls, leading to the loss of petals [4, 14]. The double flower phenotype in peach and rose is correlated with the absence of the *miR172* target site in *PETALOSA* TOE-type genes [15]. Specifically, the lack of the 10th exon in *Prupe.6G242400* in peach induces premature termination, resulting in the expression of a truncated mRNA that escapes regulation by *miR172* [15]. It is noteworthy that similar variants of *PETALOSA* TOE-type genes were found in *Rosa rugosa, Rosa Chinensis*, *Prunus persica*, *Petunia hybrida*, and *Dianthus* genus [15–19]. From the preceding discussion, it is evident that the variations in *AG* or *PETALOSA* TOE-type genes predominantly contribute to the formation of double flowers.

*Prunus mume* (Mei), a member of the Rosaceae family, has been domesticated as an ornamental plant, particularly across the entire East Asia. It is one of the earliest ornamental plants globally to undergo comprehensive studies involving whole genome assembly and resequencing [20–22]. During the long-term cultivation and selective breeding, *P. mume* has gradually developed a rich diversity of flower types, emerging as a rare material to study the genetics of floral organs in the *Prunus* genus and Rosaceae. Double flowers with excessive petals hold a significant ornamental value. The double flower trait in *P. mume* was first identified through genome wide association studies (GWAS) within a 3.6 Mb region on Chr1 [21].

To determine the cause of double-flower formation in *P. mume*, we pinpointed the candidate locus via BSA-seq and successfully identified the *PmAP2L* gene. We found that a 49-bp deletion in *PmAP2L* negatively regulated the multiple floral organ identity genes by affecting *miR172a*-*PmAP2L* interaction. Our results reveal the regulatory network of *PmAP2L* in double flower formation and provide a new perspective for studying the double-flowers in the *Prunus* genus or Rosaceae.

## Results

### Double flower morphology, development, and genetic analysis in *P. mume*

**Figure 1:**
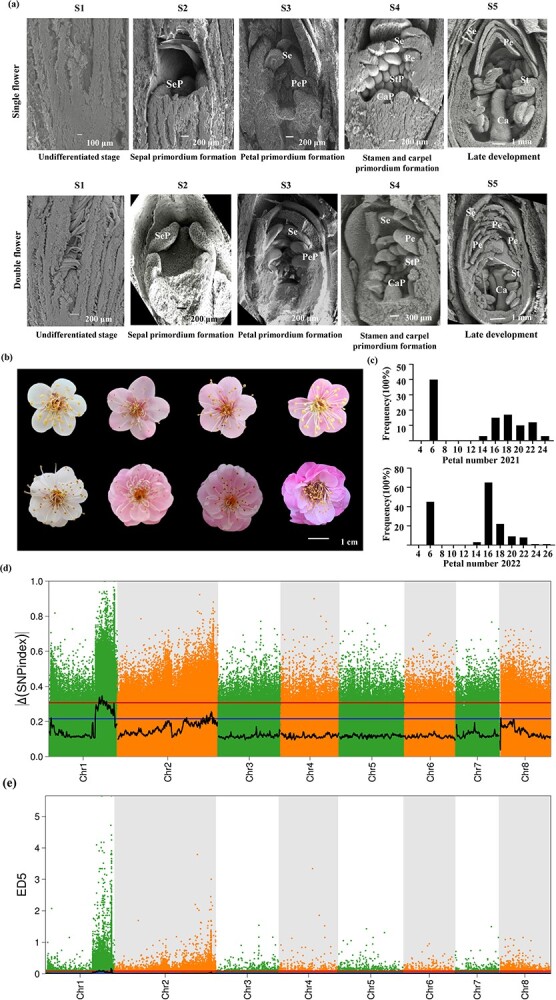
Morphology and gene locus of the double flower trait in *Prunus mume*. **(a)** SEM imaging of floral organ primordium at different developmental stages from single flower (upper panels) and double flower (lower columns). S1: undifferentiated stage; S2: sepal primordium formation; S3: petal primordium formation; S4: stamen primordium and carpel primordium formation; S5: late development. Sep, Se, PeP, Pe, StP, St, CaP, and Ca represent sepal primordium, sepal, petal primordium, petal, stamen primordium, stamen, carpel primordium, and carpel, respectively. **(b)** Flower morphology of different offsprings in the F_1_ segregation population. **(c)** Histogram of the frequency distribution of the number of petals in an F_1_ segregation population in 2021 and 2022. **(d)** Scatter diagram of the ΔSNP index. The black line indicates the fitted ΔSNP index value, and the blue and red lines represent thresholds of 99% and 95%, respectively. **(e)** Scatter diagram of ED^5. The black line indicates the fitted ED^5 value, and the red dotted line indicates the significance association threshold.

Female parent 'Mi Danlv' (MDL, single flower with five petals) and pollen donor 'Baixu Zhusha' (BXZS, double flowers with an average petal number of 24.1) were used as cross parents (Fig. S1a and Table S1, see online supplementary material). Comparison of the developmental process of single and double flower buds by SEM revealed a clear difference between the two types of buds after third stage of flower development (Fig. 1a). Double buds produced more petal primordia than single buds after differentiation of sepal primordia. As a result, at stage 5, the petal primordia in double buds completed their development into a petal sheet structure, and their number significantly exceeded that of single buds (Fig. 1a). In a blooming flower, the size of the petals gradually decreased from the outside to the inside, with clear traces of stamen petalization (Fig. S1b, see online supplementary material).

The F_1_ population in *P. mume* was generated by a cross between MDL and BXZS. The double-flower trait was particularly distinct in the F_1_ hybrids (Fig. 1b). The single-flower progeny had five petals, while the double-flower progeny had multiple layers of petals (Fig. 1b). Petal number in the F_1_ hybrid population ranged from 5 to 24 in 2021 and from 5 to 25.1 in 2022 (Fig. 1c; Table S1, see online supplementary material). The distribution of petal number was similar in both years, with a discontinuous bimodal distribution, consistent with the inheritance of qualitative traits caused by a single gene (Fig. 1c).

The major gene plus polygene mixed inheritance model was used to analyse double flower trait in 2021 and 2022. These candidate models were selected based on the minimum criteria of Akaike’s Information Criterion (AIC). The AIC values for 11 genetic models of the number of petals of F_1_ population are presented in Table S2 (see online supplementary material). A-1 and A-3 were chosen for petal number in 2021 and 2022, with the same priority order. Based on lower AIC values, the final candidate model was A-1, indicating that double flower phenotype was controlled by one-pair additive-dominant major genes. In 2021 and 2022, the additive effects, dominant effects, and major-gene heritability were as follows: 8.3290, 3.9004, and 96.1415% for 2021, and 6.8006, 3.4087, and 85.4499% for 2022, respectively (Tables S3 and S4, see online supplementary material). By comparing the frequency distribution with the theoretical distribution curve, it was found that the models for both years fitted well, and the range predicted by the model was very close to the actual distribution (Fig. S2, see online supplementary material).

### BSA-seq

To identify the key genetic locus controlling the double flower formation in *P. mume*, DNA from 64 double-flowered plants and 41 single-flowered plants was mixed and used for BAS-seq. The double and single flower pools generated a total of 112 million and 83 million high-quality raw reads, respectively (Q20 ≥ 96.34%, Q30 ≥ 91.26%) (Table S5, see online supplementary material). A total of 97.86% and 97.77% of the filtered data from the bulked samples were aligned to *P. mume* reference genome with more than 10 × depth (Table S6, see online supplementary material). Finally, 1 589 316 single nucleotide polymorphisms (SNPs) and 318 316 insertions or deletions (IN/DELs) were detected in double-flowered pool and 1 583 269 SNPs and 314 603 IN/DELS were identified in single-flowered pool (Tables S7 and S8, see online supplementary material). After eliminating the low-quality variants in two pools, 1 048 575 variants were identified across all eight chromosomes (Dataset S1, see online supplementary material).

After calculating the SNP index and the delta SNP index in the pools and plotting them against the genome positions, two candidate regions on chromosome 1 were mapped to a physical distance of 0.59 Mb from 26 720 001 to 27 310 000 bp and 3.38 Mb from 23 190 001 to 26 570 000 bp (99% confidence threshold, Fig. 1d and Table S9, see online supplementary material). In addition, two loci were also mapped on chromosome 1 by using the ED algorithm. One of the loci was located within a 0.99-Mb region from 30 180 001 to 31 170 000 bp, and the other was located within a 3.03-Mb region from 24 390 001 to 27 420 000 bp (99% confidence threshold, Fig. 1e and Table S10, see online supplementary material). The regions identified by SNP index and the ED method showed overlap. Considering the high heterozygosity of woody plants, we defined the union of the two results as the candidate interval, which was 4.23 Mb and 0.99 Mb, for subsequent analysis (Tables S9 and S10, see online supplementary material). Interestingly, the interval for the double-flower trait identified in this study partially overlapped with the region identified by our previous GWAS study [21], with a sharing of 214 genes (Dataset S2, see online supplementary material). Among these genes, there was an *AP2/ERF* transcription factor (TF), which belonged to the A-class genes and was named as *PmAP2-like* (*PmAP2L*). Additionally, both delta SNP index and the ED methods also showed a partially overlapping region on chromosomes 2 and 8 (95% confidence thresholds, Tables S11 and S12, see online supplementary material). This region was worthy to investigate the potential presence of new genes interacting with PmAP2L and influencing the formation of double flowers.

### A 49-bp deletion in *PmAP2L* affected miR172a-*PmAP2L* interaction

To confirm the potential involvement of *PmAP2L* in the formation of double flowers, we cloned the CDS of *PmAP2L* from the single and double flower individuals. Comparing with *PmAP2L-S* (*PmAP2L* from single-flowered individuals), a 49-bp deletion in the C-terminus was found in the *PmAP2L-D* (*PmAP2L* from double-flowered individuals) (Fig. S3, see online supplementary material). Furthermore, we sequenced the CDS of *PmAP2L* from other varieties with different flower types. Interestingly, the deletion was also observed in two double-flowed varieties, whereas one single-flowered did not show this deletion (Fig. S3, see online supplementary material). In addition, we cloned fragments based on the 49-bp deletion region of gDNA from the double flower pool and single flower pool of the F_1_ population, as well as from randomly selected pools of 10 single and 10 double flower varieties. Notably, the 49 bp deletion was also present in both the double-flowered progenies and double-flowered varieties compared to the single-flowered pools (Fig. S4, see online supplementary material). Moreover, a cleaved amplified polymorphic sequences (CAPS) marker was developed based on the 49-bp deletion, which was closely associated with the double flower trait (Fig. S4, see online supplementary material). The accuracy of the CAPS marker was 96.62%, suggesting that the 49-bp deletion of *PmAP2L* was closely associated with the double flower trait (Fig. S5, see online supplementary material).

**Figure 2:**
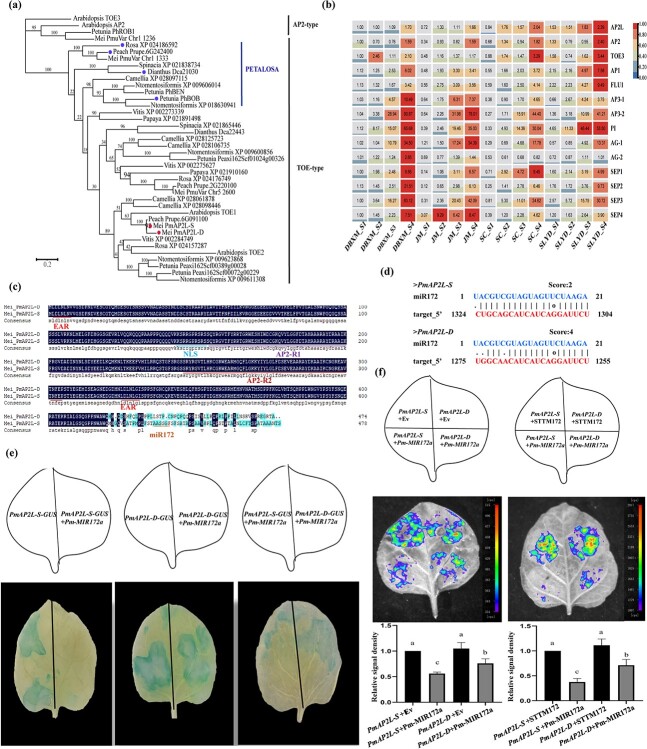
Phylogenetic and expression pattern of *PmAP2L* and the interaction of *miR172* and *PmAP2L*. **(a)** Evolutionary relationships among euAP2 (AP2- and TOE-type) members in *Prunus mume* and other species. **(b)** Expression heatmap of *PmAP2L* and other ABCDE model genes at different stages in different flower type varieties of *P. mume*. **(c)** Multiple sequence alignment analysis of PmAP2L-D and PmAP2L-S. **(d)** Prediction of *miR172* on *PmAP2L-S/D* in *P. mume*. **(e)** GUS histochemical staining. *35S::PmAP2L-S-GUS* is a vector containing *PmAP2L-S*. *35S::PmAP2L-D-GUS* is a vector containing *PmAP2L-D*. *35S::Pmu-pre-miR172a* is a vector containing mature *miR172a*. The experiments were independently repeated three times. **(f)** Validation of the miR172a-*PmAP2L* interaction *in vivo*. Empty-SK refers to the empty vectors. *35S::LUC-PmAP2L-S* is a vector containing the *miR172* target site fragments of *PmAP2L-S*. *35S::LUC-PmAP2L-D* is a vector containing the miR172 target site fragments of *PmAP2L-D*. *SK-Pm-MIR172a* is a vector containing mature *miR172a*. *SK-STTM172* is a vector containing a short tandem target mimic *miR172* fragment (*STTM172*). The experiments were independently repeated three times. The upper panels represent the mixed injection of vectors, the middle panels show representative leaf images, and the lower columns represent the statistical analyses of relative luminescence intensities. The relative luminescence intensities were calculated with the value of *PmAP2L-S* + EV combination (left bar chart) and *PmAP2L-S* + *STTM172* combination (right bar chart) as 1, respectively. Data are represented as the mean ± SD of five biological replicates (*n* = 5). Different letters above the bars indicate a significant difference (*P* < 0.05, one-way ANOVA).

Phylogenetic analysis revealed that PmAP2L belonged to the TOE-type genes and it was clustered in the same clade with Arabidopsis_TOE1, Rosa_XP_024157287, and Peach_Prupe.6G091100 (Fig. 2a). Moreover, Rosa_XP_024186592, Peach_Prupe.6G242400, Dianthus_Dca21030, and Petunia_PhBOB, which were reported to be associated with the double flower formation, were distributed in the same branch, also known as PETALOSA TOE-type. Mei_PmuVar_chr1_1333 was also distributed in this branch. The sequence alignment and conserved domain distribution suggested that the gene structures and motif arrangements were similar in both branches, but PETALOSA TOE-type genes did not contain motif 10 (Fig. S6a–c, see online supplementary material). The sequence similarity analysis showed that the similarity index among PETALOSA TOE-type genes was 59%–98%. The similarity index among Mei_PmAP2L-D/S, Arabidopsis_TOE1, Rosa_XP_024157287, and Peach_Prupe.6G091100 was 60%–99% (Fig. S6d, see online supplementary material). Moreover, the two branches were clearly distinguishable based on the domain distribution and the similarity index (Fig. S6c–d, see online supplementary material). Through microsynteny analysis between PmAP2L locus and PETALOSA TOE-type genes locus, we found that mei PmAP2L locus exhibited strong collinear relationship with rose RC5G0530900 locus on chromosome 5 and peach Prupe.6G091100 locus on chromosome 6. The rose RcAP2L (Rc5G0243000) locus and the peach Prupe.6G242400 locus showed strong collinear relationship with mei PmuVar_Chr1_1333 locus (Fig. S6e–f, see online supplementary material). These results suggested that PmAP2L was not a PETALOSA TOE-type gene. It might be a new gene controlling the formation of double flower in *P. mume*.

PmAP2L possesses several conserved domains, including the two AP2 domains (AP2-R1 and AP2-R2), nuclear localization sequence (NLS), and two typical ethylene-responsive element binding factor-associated amphiphilic repression (EAR) motifs (Fig. 2c). To further determine whether there were differences in the target sites, we used the TAPIR online tool to identify the *miR172* target site in *PmAP2L*. Interestingly, the 49 bp deletion was spanned in the *miR172* target sequence, causing sequence differences between the *miR172* cleavage site of *PmAP2L-D* and *PmAP2L-S* (Fig. 2d). The *miR172* target site differed by only two bases in the sequence of *PmAP2L-S* from single flower plants, while the double flower plants differed by four bases (Fig. 2d).

To explore the prospective function of *PmAP2L* in controlling flower development, as well as the differential regulation by *miR172*, we measured the relative expression levels of *PmAP2L* in the buds of single and double flower individuals. As shown in Fig. S7 (see online supplementary material), the expression of *PmAP2L* in the double-flower individual was significantly higher than that of the single flower individual, possibly due to the 49-bp deletion leading to a decrease in the ability of *miR172* to bind to the *PmAP2L-D.* In addition, we selected 14 genes in ABCDE model for gene expression patterns in four varieties, including single flower varieties DBXM and JM, and double flower varieties SC and SLYD (Fig. 2b). The transcript level of the A-class genes (*PmAP2L*, *PmAP2*, *PmTOE3*, *PmAP1*, and *PmFLU1*) increased gradually during floral bud differentiation (Fig. 2b). Notably, except for the undifferentiated stage (S1), the expression of *PmAP2L* was significantly high in double-flower varieties as compared to single-flowered, consistent with the results of flower buds of F_1_ population (Fig. 2b). Regarding class B genes, *PmAP3–1*, *PmAP3–2*, and *PmPI* showed a gradually increasing trend during flower development. Especially, the expression level of *PmAP3–2* and *PmPI* rose sharply after the petal primordium formation stage (S3) (Fig. 2b). *PmAG-1* and *PmAG-2* expressed during the petal primordium formation stage (S3), and the expression level was higher in single flowers as compared to double-flower varieties at S3–S4. This suggested that the expression of *PmAG-1* and *PmAG-2* was correlated with the formation and differentiation of petal primordia (Fig. 2b). The transcript of the E-class genes *PmSEP1/2/3/4* increased gradually at S1–S4, with *PmSEP3* showing the highest expression level at the primordium formation stage of stamen and carpel (Fig. 2b).

The interaction between *miR172* and *PmAP2L* was validated by GUS histochemical staining and luciferase imaging assay. Compared with *PmAP2L-D* and *PmAP2L-S* group, the decreased GUS expression in *PmAP2L-D*-GUS + *Pm-MIR172a* and *PmAP2L-S*-GUS + *Pm-MIR172a* group suggested that *miR172a* could target and degrade the mRNA of *PmAP2L-D* and *PmAP2L-S* (Fig. 2e)*.* Interestingly, the reduction in GUS expression in *PmAP2L-D*-GUS + *Pm-MIR172a* group was considerably weaker when compared to *PmAP2L-S*-GUS + *Pm-MIR172a* group (Fig. 2e). This indicated that miR172a in the double-flower alleles had a lower cleaving efficiency of *PmAP2L* than that in the single-flower alleles. The results of the LUC luminescence signal are shown in Fig. 2f. The control group of *PmAP2L-D/S*-LUC + Empty-SK and *PmAP2L-D/S*-LUC + SK-STTM172 produced a strong luminescence signal, while the signals of *PmAP2L-S*-LUC + *SK-Pm-MIR172a* and *PmAP2L-D*-LUC + SK-*Pm-MIR172a* groups were weak. These results indicated that *PmMIR172a* had an inhibitory effect on *PmAP2L-D* and *PmAP2L-S*, hindering the expression of the LUC and making the signal weaker. Importantly, the LUC luminescence signal of *PmAP2L-D*-LUC + SK-*Pm-MIR172a* group was significantly higher than that of *PmAP2L-S*-LUC + SK-*Pm-MIR172a* group, indicating that the efficiency of *miR172* in cutting the *PmAP2L* in the double-flower alleles was lower than that in the single flower alleles. The interaction between miR172 and *PmAP2L* was validated by the dual luciferase reporter assay.

### *PmAP2L-D* but not *PmAP2L-S* regulates stamen petaloids

**Figure 3:**
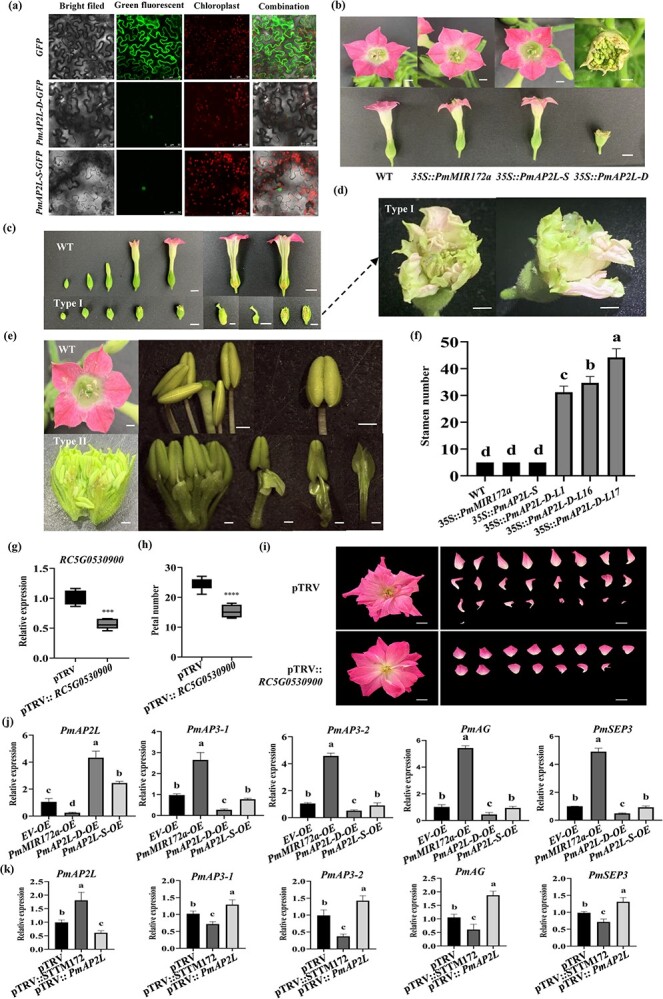
The function of *PmAP2L-D* in regulating stamens into petals. **(a)** Subcellular localization of PmAP2L-S/D in tobacco leaves. **(b)** Phenotypic observation of *35S::Pmu-pre-172a*, *35S::PmAP2L-S*, and *35S::PmAP2L-D* transgenic tobacco plants. Scale bar = 1 cm. **(c)***35S::PmAP2L-D* tobacco (type I) show the formation of two rounds of floral structures within the sepals, forming another corolla within the corolla. Scale bar = 1 cm. **(d)** Amplification of the type I phenotype. Scale bar = 1 cm. **(e)***35S::PmAP2L-D* tobacco (type II) show the transformation of stamens to petals. Scale bar = 1 cm. **(f)** The number of stamens in transgenic tobacco with *miR172a*, *PmAP2L-S*, and *PmAP2L-D* genes. Data are represented as the mean ± SD of five biological replicates (*n* = 5). Different letters above the bars indicate a significant difference (*P* < 0.05, one-way ANOVA). **(g)** qRT-PCR of *RC5G0530900* in TRV control and silenced plants. The mean ± SD from three biological replicates (*n* = 3) are shown. Asterisks indicate statistically significant differences (two-sided Student's *t* test; ^***^P < 0.001). **(h)** The number of petals in TRV and TRV-*RC5G0530900*. The mean ± SD from 12 biological replicates (*n* = 12) are shown. Asterisks indicate statistically significant differences (two-sided Student's t test; ^****^P < 0.0001). **(i)** Images of TRV and TRV- *RC5G0530900* infected plants were taken 50 d after infiltration. **(j)** qRT-PCR of *PmAP2L* and floral development genes (*PmAP3–1*, *PmAP3–2*, *PmAG*, and *PmSEP3*) in flower buds after *PmAP2L-D/S* and *PmMIR172a* overexpression. EV, empty vector; OE, overexpression. The mean ± SD from three biological replicates (*n* = 3) are shown. Different letters above the bars indicate a significant difference (*P* < 0.05, one-way ANOVA). **(k)** qRT-PCR of *PmAP2L* and floral development genes (*PmAP3–1*, *PmAP3–2*, *PmAG*, and *PmSEP3*) in flower buds after VIGS treatment. The mean ± SD from three biological replicates (*n* = 3) are shown. Different letters above the bars indicate a significant difference (*P* < 0.05, one-way ANOVA).

The results of subcellular localization showed that the green fluorescent protein (GFP) signal of the control group was detected in the nucleus and cell membrane, while PmAP2L-D and PmAP2L-S were only localized to the nucleus (Fig. 3a).

In order to verify the functions of *PmAP2L-D/S* and *miR172* in the regulation of flower organ development, we overexpressed the Pmu-pre-miR172a (miR172-OE), *PmAP2L-S* (PmAP2L-S-OE), and *PmAP2L-D* (PmAP2L-D-OE) in tobacco leaves by *Agrobacterium*-mediated transformation. The quantitative real-time (qRT)-PCR analysis showed that the expression levels of *miR172a*, *PmAP2L-S*, and *PmAP2L-D* in the leaves of transgenic lines were higher than those in WT. The expressions of *miR172a*, *PmAP2L-S*, and *PmAP2L-D* were increased by more than 150, 50, and 300-fold, respectively, in the severely affected transgenic plants (Fig. S8, see online supplementary material). Phenotypically, all miR172a-OE and PmAP2L-S-OE transgenic plants showed no significant difference in flower color, stamen, pistil, and other traits compared to WT plants (Fig. 3b). However, three of the PmAP2L-D-OE plants (OE1, OE16, and OE17) were found to have small flower organs and light flower color, along with an increase in the number of stamens, reaching up to 44 stamens (Fig. 3c–f). The PmAP2L-D-OE lines had two major types of stamens turned into petals with downregulation ofendogenous genes related to flower development (Fig. S9, see online supplementary material). Type I transgenic lines showed smaller flower organs and shorter corollas compared to WT (Fig. 3c). The flower organs exhibited incomplete development, as vertical sections revealed the formation of two rounds of floral structures within the sepals. This resulted in the creation of an additional corolla within the original corolla, with stamens and pistils still present, showing incomplete development of the flower organs (Fig. 3c–d). Type II transgenic lines showed an increase in the number of stamens, with some stamens becoming smaller, and the transformation of stamens to petals (Fig. 3e). These results indicated that the 49-bp deletion reduced post-transcriptionally the regulation of *PmAP2L-D* by miR172.

Furthermore, we selected rose, which is closely related to *P. mume* in the Rosaceae family, to further characterize the *AP2L* and determine whether knockdown of *AP2* homologs would reduce petal number. Using the same sequence (78 amino acids) shared between *PmAP2L* and the rose homologue *RC5G0530900* (Fig. S10, see online supplementary material), a VIGS vector was constructed to reduce the expression level of *RC5G0530900*. Compared with TRV-only controls, the expression level of *RC5G0530900* was decreased by 34% in *TRV-RC5G0530900* lines, and the petal number of *RC5G0530900*-silenced plants was significantly decreased (Fig. 3g–i).

**Figure 4:**
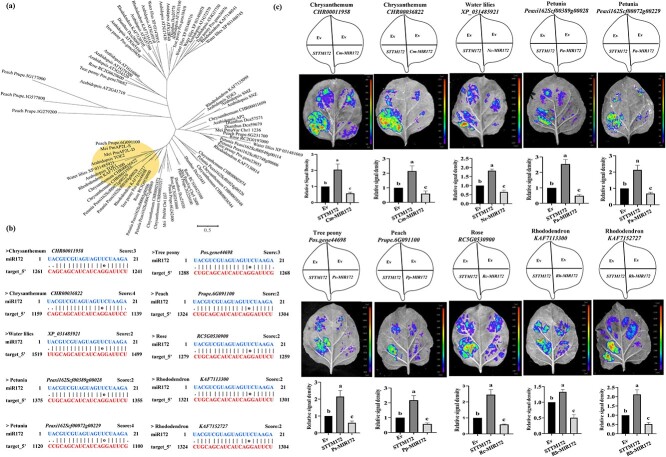
Analysis of *miR172* regulation on *AP2L* in various ornamentals. **(a)** Phylogenetic tree analysis of AP2Ls in plants. **(b)** Prediction of miR172 on *AP2L* homologs in other ornamental plants. **(c)** Validation of the *miR172*-*AP2L* interaction in different ornamental plants. The upper panels represent the mixed injection of vectors, the middle panels show representative leaf images, and the lower columns represent the statistical analyses of relative luminescence intensities. The relative luminescence intensities were calculated with the value of EV combination as 1, respectively. Data are represented as the mean ± SD of five biological replicates (*n* = 5). Different letters above the bars indicate a significant difference (*P* < 0.05, one-way ANOVA).

To further verify the function of *PmAP2L-D/S* and miR172 in the regulation of floral organ development, we generated *PmAP2L* and *Pmu-pre-miR172a* overexpression and silencing vectors. The vectors were transferred into *P. mume* flower buds using agroinfiltration. qRT-PCR results showed that the expression of *PmAP2L* was significantly increased after the overexpression of *PmAP2L-D/S* and silencing of *PmMIR172* (Fig. 3j and k). In contrast, the expression of *PmAP2L* was significantly reduced after the overexpression of *PmMIR172* and silencing of *PmAP2L* (Fig. 3j and k). Subsequently, the expression levels of floral development genes (*PmAP3–1*, *PmAP3–2*, *PmAG*, and *PmSEP3*) were reduced after the overexpression of *PmAP2L-D/S* and silencing of *PmMIR172*. Contrarily the expression levels of these genes were increased after the overexpression of PmMIR172 and silencing of *PmAP2L* (Fig. 3j and k). Notably, overexpression of *PmAP2L-D* inhibited the expression of *PmAP3–1*, *PmAP3–2*, *PmAG*, and *PmSEP3* genes more significantly than that of *PmAP2L-S* (Fig. 3j and k). Thus, these results suggested the functional differences between *PmAP2L-D* and *PmAP2-S* in the regulation of flower development in *P. mume*.

### *mi172*-*AP2L* module regulates the double-flower trait in various ornamentals

To examine the potential conservation of the *miR172*-*AP2L* module in regulating flower organ development, *AP2* genes from eight ornamental plants (petunia, rose, peach, tree peony, water lily, rhododendron, carnation, and chrysanthemum) and Arabidopsis were selected to construct a phylogenetic tree (Fig. 4a; Fig. S11, see online supplementary material). It was found that except for carnations, other plants had AP2L homologous genes that were clearly clustered with mei PmAP2L-D/S and AtTOE1 (Fig. 4a, Fig. S10, see online supplementary material). Bioinformatics predictions found that the subfamily of AP2L related to the double-flower trait had high sequence complementarity with *miR172*, and the homology of proteins from different ornamentals was high (Fig. 4b). Furthermore, LUC results showed that *miR172* repressed the expression of homologs of *AP2L* (Fig. 4c). Thus, the *miR172-AP2L* module appears to be conserved in regulating the double-flower phenotype in various ornamentals. The interaction between *miR172* and *AP2L* in different ornamentals was validated by the dual luciferase reporter assay.

### The 49-bp variation of *PmAP2L* affects its binding ability to floral organ identity genes

**Figure 5:**
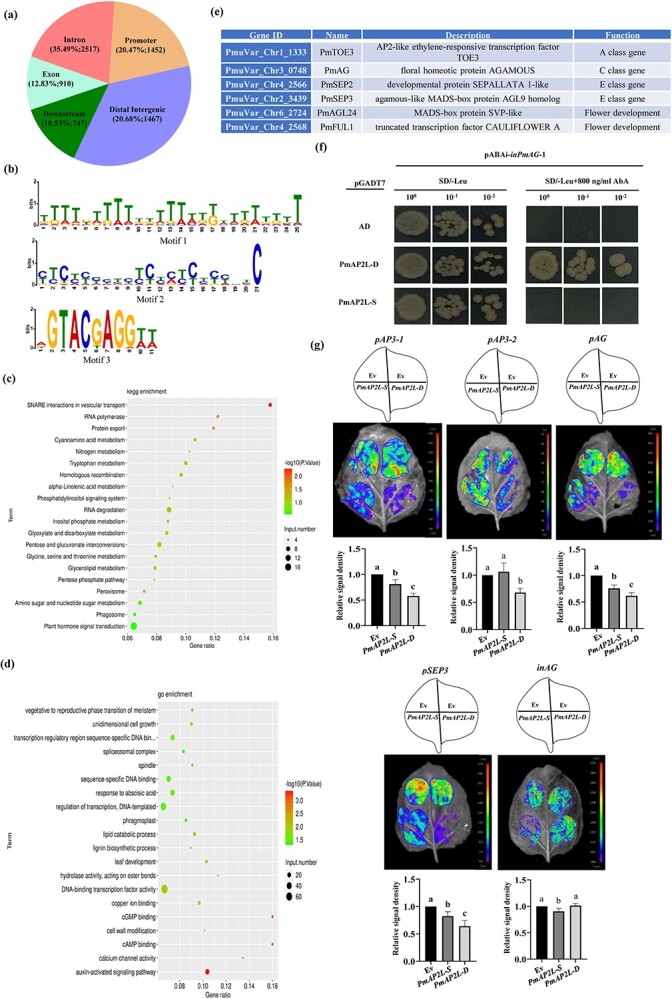
A 49-bp variation of *PmAP2L* affects its binding ability to floral organ identity genes. **(a)** Distribution of PmAP2L-D binding regions in *Prunus mume* genome based on DAP-seq analysis. **(b)** Conserved motifs of PmAP2L-D identified from the DAP-seq data. **(c)** KEGG enrichment analysis of all putative target genes regulated by PmAP2L. **(d)** GO enrichment analysis of all putative target genes regulated by PmAP2L. **(e)** PmAP2L may directly regulate genes related to flower development. **(f)** Y1H assay showing that the PmAP2L-D can bind directly to the second intron of *PmAG*, while PmAP2L-S cannot bind. **(g)** Effect of PmAP2L-D/S on the activity of the *PmAP3–1*, *PmAP3–2*, *PmAG*, and *PmSEP3* promoters and the second intron of *PmAG* by dual-luciferase assay. The upper panels represent the mixed injection of vectors, the middle panels show representative leaf images, and the lower columns represent the statistical analyses of relative luminescence intensities. The relative luminescence intensities were calculated with the value of EV combination as 1, respectively. Data are represented as the mean ± SD of five biological replicates (*n* = 5). Different letters above the bars indicate a significant difference (*P* < 0.05, one-way ANOVA).

To determine the regulatory function of *PmAP2L* in the flower development, DAP-seq was used. A total of 7093 *PmAP2L-D* binding sites were identified, corresponding to 5760 genes (Dataset S3, see online supplementary material). Among them, 35.49% (2517 peaks), 20.68% (1467 peaks), 20.47% (1425 peaks), 12.83% (910 peaks), and 10.53% (747 peaks) were located in intronic, distal intergenic, promoter, exon, and downstream regions, respectively (Fig. 5a; Dataset S3, see online supplementary material). Through MEME-CHIP analysis of the binging peaks, the most significantly enriched motif sequence distribution was TTTT(G/T)TTTTT(G/T)TTT(A/T)T(G/T)(A/T)TTTTTTT，occupying approximately 63.26% of the peaks (Fig. 5b). GO enrichment analysis of these potential PmAP2L target genes showed that they were mainly enriched in DNA-binding transcription factor activity, regulation of transcription, DNA-templated, and sequence-specific DNA binding, while the auxin-activated signaling pathway had the lowest *P* value (Fig. 5c). KEGG analysis annotated the largest number of genes in plant hormone signal transduction pathway (Fig. 5d). Among these potential target genes, many genes related to flower development in the ABCDE model were found, such as class A gene *PmTOE3*, class C gene *PmAG*, class E gene *PmSEP2/3*, as well as flowering-related genes *PmAGL24* and *PmFUL1* (Fig. 5e; Dataset S3, see online supplementary material).

To confirm whether PmAP2L can regulate ABCDE model genes and to explore whether the 49-bp variation in *AP2L* affects its regulatory efficiency on ABCDE model genes, we performed yeast one-hybrid (Y1H) and luciferase complementation assay. First, through Y1H assays, it was found that except for the second intron of *PmAG*, other genes’ promoters were self-activated and could not be further tested. Y1H results showed that yeast cells containing either pGADT7-*PmAP2L-D/S* or pGADT7-empty grew well on non-selective medium SD/−Leu. Only the bait yeast cells co-transformed with pGADT7-*PmAP2L-D* survived on selective medium SD/−Leu/ + 800 ng/mL AbA (Fig. 5f). This indicated that the PmAP2L-D might bind directly to second intron of *PmAG*, while PmAP2L-S might not bind.

To detect if the PmAP2L-D and PmAP2L-S can inhibit the expression of ABCDE model genes in plants, we co-expressed *35S::PmAP2L-D/S* vectors (effectors) with *pPmAP3–1::LUC*, *pPmAP3–2::LUC*, *pPmAG::LUC*, *pPmSEP3*:*:LUC*, and *inPmAG::LUC* (reporters) in dual-luciferase reporter assays. The activity of the *PmAP3–1*, *PmAG*, and *PmSEP3* promoters fused with the *LUC* gene was significantly inhibited by PmAP2L. Here, PmAP2L-D inhibited the LUC activity stronger than PmAP2L-S (Fig. 5g). Overexpression of *PmAP2L-S* had no significant effect on the LUC activity driven by the *PmAP3–2* promoter compared to empty vector, while LUC activity was significantly depressed by the expression of *PmAP2L-D* (Fig. 5g). Conversely, compared to using the empty vector, PmAP2L-D had no significant inhibitory effect on the LUC activity driven by the second intron of *PmAG*, while PmAP2L-S significantly inhibited the LUC activity of *inPmAG* (Fig. 5g). The effect of PmAP2L-D/S on the activities of the *PmAP3–1*, *PmAP3–2*, *PmAG*, and *PmSEP3* promoters and the *PmAG* second intron was validated by the dual luciferase reporter assay.

### PmAP2L interacts with the PmTPL and PmHDA6/19

**Figure 6:**
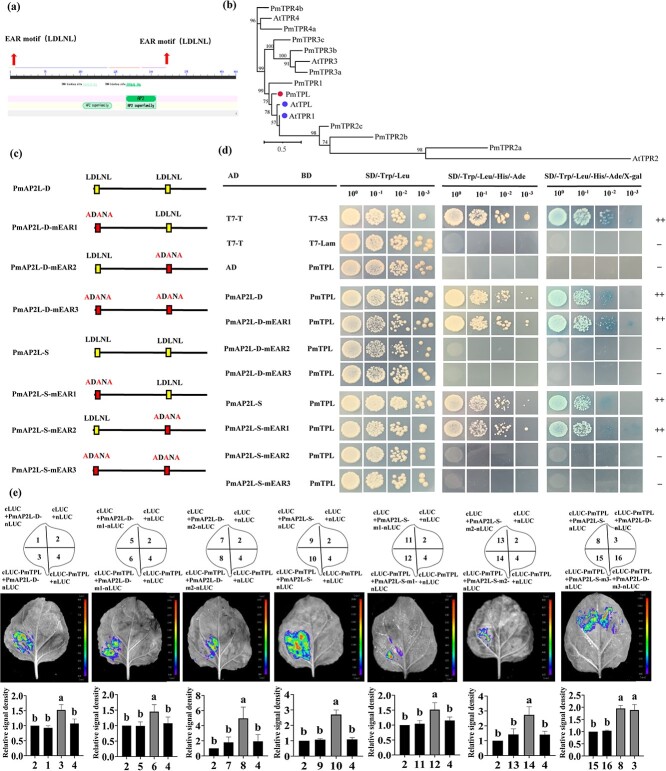
PmAP2L interacts with the PmTPL **(a)** Schematic representation of the PmAP2L protein structure. **(b)** Phylogenetic analysis of the TPL/TPR proteins. **(c)** Mutation of the EAR motif (mEAR) in the PmAP2L-D/S protein sequence. **(d)** Y2H assays showing that PmAP2L-D/S interacts with PmTPL through the EAR2 motif **(e)** LCI assays showing the interaction between PmTPL and PmAP2L through the EAR1 and EAR2 in tobacco leaves. The upper panels represent the mixed injection of vectors, the middle panels show representative leaf images, and the lower columns represent the statistical analyses of relative luminescence intensities. The relative luminescence intensities were calculated with the value of combinations 2, 2, 2, 2, 2, 2, 8 as 1, respectively. Data are represented as the mean ± SD of five biological replicates (*n* = 5). Different letters above the bars indicate a significant difference (*P* < 0.05, one-way ANOVA).

Proteins harboring EAR motifs play a crucial role in diverse biological processes by suppressing target gene expression. Transcription factors containing the EAR motif were reported to interact with TOPLESS (TPL) corepressor. The PmAP2L contained two typical EAR motifs (LxLxL), EAR1 and EAR2, located in the N-terminal and middle regions, respectively (Fig. 6a). We identified the mei TPL/TPR protein by phylogenetic analysis and named as PmTPL (PmVar_chr4_0936), which was close to AtTPL from the Arabidopsis (Fig. 6b). In order to explore whether PmAP2L-D/S functions as transcriptional repressors, the EAR motifs in PmAP2L-D/S were mutated and named as PmAP2L-D-mEAR1/2/3 and PmAP2L-S-mEAR1/2/3, respectively (Fig. 6c). Y2H assays between PmAP2L-D/S and PmTPL were performed and the results showed that PmA2PL-D/D-mEAR1 and PmA2PL-S/S-mEAR1 interacted with PmTPL, whereas mutation of the EAR2 motif in PmAP2L-D/S failed to do so (Fig. 6d). This suggested that the EAR2 motif rather than EAR1 was crucial for the interaction between PmAP2L-S/D and PmTPL. Luciferase complementation imaging (LCI) assays were performed and the results showed that the EAR3 (PmA2PL-D-mEAR3 and PmA2PL-S-mEAR3) abolished the interaction between PmAP2L-D/S and PmTPL (Fig. 6e). This indicated that the EAR was required for PmAP2L to interact with PmTPL.

**Figure 7:**
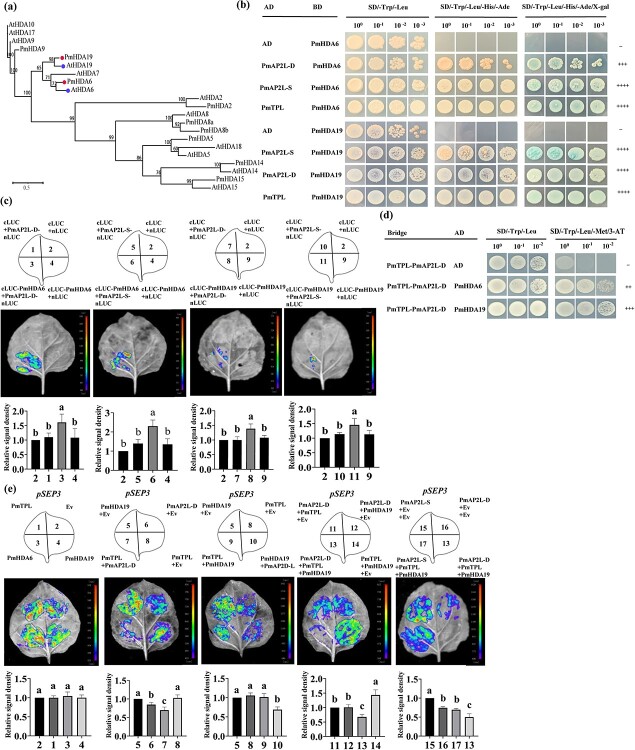
The 49-bp variation of *PmAP2L* affects PmAP2L-PmTPL-PmHDA19 binding ability to floral organ identity genes. **(a)** Phylogenetic analysis of PmHDA6/19 with HDAs from *Arabidopsis thaliana*. **(b)** PmAP2L interacts with the PmTPL and PmHDA6/19 by Y2H assay. **(c)** LCI assays showing the interaction between PmHDA6/19 and PmAP2L-D/S in tobacco leaves. The upper panels represent the mixed injection of vectors, the middle panels show representative leaf images, and the lower columns represent the statistical analyses of relative luminescence intensities. The relative luminescence intensities were calculated with the value of combination 2 as 1, respectively. Data are represented as the mean ± SD of five biological replicates (*n* = 5). Different letters above the bars indicate a significant difference (*P* < 0.05, one-way ANOVA). **(d)** Y3H was carried out to detect the interaction among PmAP2L, PmTPL, and PmHDA6/19. **(e)** Dual-luciferase assay showed that the activity of the *PmSEP3* promoters fused with the *LUC* gene was significantly inhibited by PmAP2L-D-PmTPL-PmHDA19 complex compared with PmAP2L-S-PmTPL-PmHDA19 complex. The upper panels represent the mixed injection of vectors, the middle panels show representative leaf images, and the lower columns represent the statistical analyses of relative luminescence intensities. The relative luminescence intensities were calculated with the value of combinations 2, 5, 5, 11, 15 as 1, respectively. Data are represented as the mean ± SD of five biological replicates (*n* = 5). Data are represented as the mean ± SD of five biological replicates (*n* = 5). Different letters above the bars indicate a significant difference (*P* < 0.05, one-way ANOVA).

Histone deacetylases (HDAs) are additional components of TPL-dependent transcriptional repression complex. HDAs actively repress gene expression in many eukaryotes, including Arabidopsis HDA6 and HDA19 [23]. Y2H and LCI assays showed that PmAP2L-D/S and PmTPL interacted with PmHDA6/19, respectively (Fig. 7a–c; Fig. S12, see online supplementary material). The interaction between PmTPL and PmAP2L and between PmHDA6/19 and PmAP2L-D/S was validated by the firefly luciferase complementation imaging assay.

### The 49-bp variation of PmAP2L affects PmAP2L-PmTPL-PmHDA19 binding affinity to floral organ identity genes

The interactions among PmAP2L-D/S, PmTPL, and PmHDA6/19 were verified by Y2H assays. Our prior analyses indicated that HDAs were involved in TPL-dependent transcriptional regulation. To determine whether the PmAP2L-TPL complex recruits HDAs to repress the expression of floral development identity genes, a Y3H assay was performed to study the interaction between the PmAP2L-TPL complex and PmHDA6 and PmHDA19. Yeast cells containing Bridge-PmTPL-PmAP2L-D and AD-PmHDA6/19 constructs grew well on SD/−Trp/−Leu and SD/−Trp/−Leu/−Met/3-AT medium (Fig. 7d), suggesting that PmAP2L-D, PmTPL, and PmHDA6/19 apparently formed a tripartite complex.

To examine whether PmAP2L recruits PmTPL and PmHDA19 to the promoter of *PmAP2L* target genes, a dual-luciferase reporter assay was performed. PmTPL, PmHDA6, and PmHDA19 barely influenced the activity of the LUC reporter. The LUC activity was significantly decreased only in the presence of PmAP2L-D, especially in the PmAP2L-PmTPL-PmHDA19 complex (Fig. 7e), suggesting that PmAP2L-D, PmTPL, and PmHDA19 might form a tripartite complex to repress the expression of floral development identity genes. In addition, compared with PmAP2L-S-PmTPL-PmHDA19 complex, our results showed that the activity of the *PmSEP3* promoters fused with the *LUC* gene was significantly inhibited by PmAP2L-D-PmTPL-PmHDA19 complex (Fig. 7e). Therefore, we proposed that the 49 bp in *PmAP2L* affected the binding of the PmAP2L-PmTPL-PmHDA19 complex to floral organ identity genes, leading to double flower formation. The interaction of PmAP2L recruiting PmTPL and PmHDA19 to the *PmSEP3* promoter was verified by the firefly luciferase complementation imaging assay.

## Discussion

The double-flower trait holds significant ornamental value. Variations in TOE-type *AP2* genes causing the double-flower phenotype have been reported in *R. rugosa*, petunia, carnation, and peach [15–17]. All the genes identified in these species belong to a single PETALOSE TOE-type subgroup, suggesting a robust conservation of the double flower phenotype throughout the course of evolution. However, the likelihood of differences between various plants and the appearance of new A class genes remains to be elucidated. The predominant focus in current research has been on identifying gene locations, with less emphasis on exploring the regulatory mechanisms governing these genes. Here, we used BSA analysis to identify a new gene, *PmAP2L*, which contributes to double-flower trait. It has a 49 bp variation that affects its binding with *miR172*. Meanwhile, we demonstrate that PmAP2L, an AP2 subfamily member containing EAR motifs, negatively regulates the transcription of multiple floral organ identity genes by forming a repressor complex with PmTPL and PmHDA6/19. We further elaborate that a 49-bp deletion in *PmAP2L* affects this regulatory process.

### *PmAP2L* is a new gene that controls the double-flower trait in *P. mume*

The AP2 TFs play critical roles in plant developmental transitions [24–26]. In Arabidopsis, there are six members of the AP2-like gene family, including *AP2*, *TARGET OF EAT1* (*TOE1*), *TOE2*, *TOE3*, *SCHLAFMUTZE* (*SMZ*), and *SCHNARCHZAPFEN* (*SNZ*) [25]. The AP2-type genes, *AP2* and *TOE3*, are necessary for cell maintenance and specification of floral organ identity [24, 27, 28], whereas the TOE-type genes, *TOE1*, *TOE2*, *SMZ*, and *SNZ*, regulate flowering time [27, 29]. In this study, we found that *PmuVar_Chr1_1333*, *Rosa_XP_024186592*, *Prupe.6G242400*, *Dianthus_Dca21030*, and *PhBOB* belong to a single TOE-type, which is also named as *PETALOSA* [16]. The *PETALOSA* TOE-type genes are involved in the formation of double-flower in many plant species, such as peach, rose, carnation, and petunia [15–17, 24, 28]. Interestingly, PmAP2L-D/S, Rosa_XP_024157287, Peach_Prupe.6G091100, AtTOE1, and AtTOE2 also belong to the TOE-type, but are not PETALOSA TOE-type (Fig. 2a; Fig. S6 and Tables S13 and S14, see online supplementary material). Moreover, the two branches can be clearly distinguished based on the domain distribution, similarity, and microsynteny analysis (Fig. S6c–f). The genes in these branches have not been reported for their role in the regulation of flower development, especially the double flower formation [30]. We speculate that *PmAP2L* is a novel gene that determines the formation of double flowers in *P. mume*. The primary evidence comes from the transgenic tobacco experiments, wherein the transgenic lines show an increase in the number of stamens and the transformation of stamens to petals (Fig. 3b–e). Indirect evidence is that *PmAP2L* homologs are directly associated with the petal numbers in rose. Reducing the transcription level of *RC5G0530900* in roses through VIGS significantly decreases the number of petals (Fig. 3h–j). Therefore, these results collectively support a new function of *PmAP2L* in floral organ identity.

### A conserved role for the miR172-AP2L pathway on the double flower formation trait in various ornamentals

The AP2 TF family, including *AP2*, *TOE1*, *TOE2*, *TOE3*, *SNZ*, and *SMZ*, are regulated post-transcriptionally by *miR172* [25, 26]. The *miRNA172*-mediated repression of *AP2* plays a central role in floral induction and flowering [25, 27]. GUS staining and luciferase reporter assays confirmed that the presence of *miR172* caused a significant reduction in GUS activities or luciferase activities driven by *PmAP2L-D/S* (Fig. 2e–f). These findings validate the regulation of *PmAP2L* by miR172. Notably, miR172 shears *PmAP2L-S* and *PmAP2L-D* differently, with miR172 repressing the single petal gene *PmAP2L-S* more significantly. Compared to wild type plants, there was no significant difference in the heterologous expression of *PmAP2L-S* in tobacco (Fig. 3b–e), while the heterologous expression of *PmAP2L-D* resulted in the transformation of stamens to petals. These results indicate that the 49 bp deletion-mediated *miR172* target site loss in *PmAP2L* has a vital role in the double flower formation.

In Arabidopsis, *AtTOE3* overexpression had no obvious phenotype. However, the overexpression of an *miR172*-resistant *TOE3* gene resulted in an indeterminate floral structure [28]. In addition, mutation of the *miR172* target site of tobacco *PET* genes resulted in double flowers [16]. These results further support the role of *miR172* target site loss in *PmAP2L* in the double flower formation. Although the roles of *miR172* or *AP2-like* have been reported in regulating flower development [27, 28], the *PmAP2L* clusters into a different subgroup from the currently reported *AP2* TFs. However, the function of *AP2-like* in this branch has not been demonstrated in the double flower formation. This provides an important reference gene for the study of double flowers in other ornamentals.

The *miR172* is a conserved microRNA, and the majority of *miR172*-*AP2* studies have centered around flower development and floral transition [24, 25]. Previous studies reveal that *AP2-like* TFs are extremely important for floral organ development [24, 27]. As an upstream regulatory factor of *AP2* transcription factors, *miR172* can eliminate the influence of *AP2-like* TFs on flower development [27, 28]. Given the importance of *miR172* and the conserved influence of *AP2-like* on flower development, it is crucial to investigate the conserved role of *miR172*-*AP2* in floral organ development. It has been partially confirmed in seven ornamental plants (petunia, rose, peach, tree peony, water lily, rhododendron, and chrysanthemum) (Fig. 4a–c). In these ornamental plants, *miR172* significantly inhibited the *AP2-like* TFs *in vivo*. Therefore, the *miR172*-*AP2L* pathway appears to contribute to the double-flower phenotype in various ornamentals.

### PmAP2L interacts with PmTPL and recruits PmHDA proteins to negatively regulate the transcription of multiple floral organ identity genes

Despite the role of miR172-mediated repression of *PmAP2L* in the formation of double flower, it is important to know whether *PmAP2L* has the A-class gene function and whether the 49-bp variants affect its function. In Arabidopsis, *TOE1* directly suppresses *FT* expression and flowering by binding to the promoter of *FT* and inhibiting the *CO* transcriptional activation activity [31]. The *TOE1* homologs, *ZmRAP2.7* in maize and *AP2L1* in wheat, are also known as suppressors of flowering [32, 33]. Unlike them, the *TOE1* homologs, *PmAP2L* in *P. mume* and *RC5G0530900* in rose, has negligible impact on the timing of flowering. Notably, overexpression of *PmAP2L-D* in tobacco produces flowers with similar morphology to those of *35S::AP2m1* lines in Arabidopsis (Fig. 3c–e), indicating that *PmAP2L* also functions as an A-class gene [27]. Consistent with this, *AG* and *SEP3* homologs, that are repressed by *AP2* in Arabidopsis, are up-regulated in single-flower *P. mume* and wild-type tobacco (Fig. 2b; Fig. S9, see online supplementary material). Contrarily, these homologs are down-regulated in double-flower *P. mume* and in tobacco overexpressing the *PmAP2L* gene [23, 24]. In addition, DAP-seq also identified multiple genes involving flower development (Fig. 5e). These results suggest that *PmAP2L* and *AtAP2* may specify floral organ identity through similar mechanisms.

Since the overexpression of *PmAP2L-D* was more effective in double flower formation compared to *PmAP2L-S*, we hypothesized that there might be some differences in the A-class gene functions between PmAP2L-D and PmAP2L-S. Here, we found that the 49-bp variance of *PmAP2L* affected its binding ability to floral organ identity genes and it negatively regulated the expression of *PmAP3*, *PmAG*, and *PmSEP3* (Fig. 5g). *PmAP2L-D* was more significant in inhibiting flower development-related genes than *PmAP2L-S*. Although *AP2-like* gene has been reported to potentially affect *AG* expression and plays a role in double flower formation in some species, such as carnation, peach, and rose [15–17, 34], these studies are mostly speculative and have no direct evidence. In our study, *PmAP2L-S* became weaker in its ability to bind to the promoter and second intron of *PmAG*, whereas *PmAP2L* showed an enhanced ability to bind to the promoter and second intron of *PmAG*, and inhibited the expression of *PmAG* more significantly (Figs 2b and 5f and g). These results imply that the 49 bp variation in *PmAP2L* leads to differences in the regulation of *AG* gene, acting as a direct cause of double flower formation in *P. mume*.

The AP2 can act both as a transcriptional activator and a direct repressor [24]. We find that PmAP2L contains two conserved EAR motifs known to repress the transcription of downstream genes [23, 30, 35]. PmAP2L-D can inhibit the expression of floral organ identity genes, *PmAP3–1*, *PmAP3–2*, *PmAG*, and *PmSEP3* by binding to their promoters. This repressor activity is gradually enhanced by the upregulation of the *PmAP2L* after the start of flower development in double flower, causing the continuous downregulation of genes such as *AG* and *SEP3* in the second and third rounds of flower organ development (Figs 2b and 5f and g), which favors double flower formation in *P. mume*. The double flower formation in ornamental plants like *P. mume* is genetically programmed and involves complex interactions between multiple plant developmental factors. Whilst the specific underlying mechanisms are not yet clear, it is generally believed that both inhibitors and activators are at work [23, 24].

**Figure 8:**
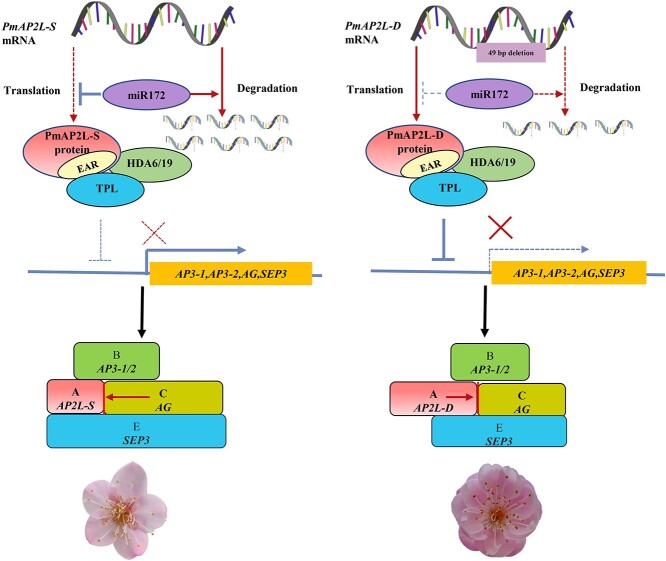
Proposed model for PmAP2L function in the formation of double flower in *Prunus mume*. In the absence of *PmAP2L* mutations, *PmAP2L-S* is more significantly regulated by *miR172* than *PmAP2L-D*. The protein encoded by PmAP2L interacts with PmTPL and PmHDA19 to form a complex, which has a weaker inhibitory effect on flower development-related genes, resulting in a significant increase in genes expression and the single-flower phenotype in *P. mume*. When *PmAP2L* has the 49 bp variant, the regulation of *PmAP2L-D* by *miR172* decreases. *PmAP2L-D* is highly expressed, and it forms an inhibitory complex with PmTPL and PmHDA19, which has a more significant inhibitory effect on flower development-related genes in the second and third floral organs, resulting in the appearance of double flower in *P. mume*.

The *AP2* containing the EAR motif in Arabidopsis has recently been proven to negatively regulate floral homeotic genes by recruiting TPL and HDA19 [23]. Here, we demonstrate that PmAP2L in *P. mume* interacts with the co-repressor PmTPL through the EAR motif to recruit the histone deacetylase PmHDA19 to form a complex that negatively regulates floral organ identity genes. Interestingly, compared with PmAP2L-S, PmAP2L-D has a more significant inhibitory effect on the expression of floral organ identity genes when forming a complex with PmTPL and PmHDA19. In summary, these data support a model where PmAP2L regulates the formation of double flower in *P. mume* (Fig. 8). *PmAP2L* is negatively regulated by miR172. In double flower, a 49 bp deletion in *PmAP2L* induces a decrease in the ability of miR172 to repress its expression, resulting in an increase in *PmAP2L-D* expression compared to *PmAP2L-S*. PmAP2L interacts with PmTPL and PmHDA19 to form a complex that inhibits the transcription of flower development-related genes. When *PmAP2L* is not mutated, PmAP2L-S becomes too weak to repress the promoters of *PmAP3–1*, *PmAP3–2*, *PmAG*, and *PmSEP3*. This results in a significant increase in genes expression and the single-flower phenotype in *P. mume*. A 49 bp deletion in *PmAP2L* changes its function and it binds to the second intron of *PmAG*, resulting in enhanced repression of *PmAG* expression. At the same time, its inhibitory effect on *PmAP3–1*, *PmAP3–2*, *PmAG*, and *PmSEP3* promoters becomes more intense, resulting in the double flower trait in *P. mume*. Therefore, our work reveals that the 49 bp variation in *PmAP2L* leads to differences in the regulation of flower development-related genes, including *PmAP2L-D* and *PmAP2L-S*. The discovery of *PmAP2L* provides valuable insights into complex regulatory pathways underlying the floral diversity of ornamental plants and sets new directions for future molecular breeding of *P. mume*.

## Materials and methods

### Plant materials

An F_1_ population of 162 individuals generated from the female parent 'Mi Danlv' and the pollen donor 'Baixu Zhusha' was used to identify the genetic locus controlling the double-flower formation in *P. mume*. The plants were cultivated in Jiangle State-owned Forest Farm, Fujian Province, China. The genomic DNA from young leaves of 64 double-flowered individuals and 41 single-flowered individuals was used for BSA-seq. *Nicotiana benthamiana*, *Nicotiana tabacum*, *R. chinensis* ‘Old Blush’ were grown as described previously [36, 37].

### Scanning electron microscopy

Floral tissues were collected every 14 d from mid-August to mid-November 2022 and stored in 2.5% glutaraldehyde solution. The flower buds were fixed at 4°C for 24 h or longer, dehydrated for 20 min through a series of alcohol and isoamyl acetate, dried at critical-point, mounted on the scanning electron microscopy (SEM) platform, and coated with gold. Finally, the buds were observed by a S-3400N scanning electron microscopy (Hitachi, Tokyo, Japan).

### Phenotyping and data analysis

The double flower phenotypes of F_1_ hybrids were ascertained during the flowering periods from January to February of 2021 and 2022, respectively. The double flower trait was assessed by the average petal number of five or more flowers in each plant. When the number of petals is 5–7, it is considered a single flower, and when the number is 15 or more, it is considered a double flower. The descriptive statistical analysis and frequency distribution histogram were performed using Microsoft Office EXCEL 2019 software. Considering that *P. mume* is a woody flower with a highly heterozygous background, and that there is obvious segregation of traits such as petal number and flower color in the F_1_ hybrids, the phenotypic data of the F_1_ hybrids were considered as pseudo F_2_ hybrids for genetic analysis. The SEA package (https://cran.r-project.org/web/packages/SEA/index.html) in R (https://www.r-project.org/) was used to analyse the major gene plus polygene mixed inheritance model in this study. Specific genetic model analysis was described with reference to the description of SEA software.

### BSA sequencing and analysis

Genomic DNA from young leaves was isolated using DNAsecure Plant Kit (Tiangen Biotech, Beijing, China). The extracted DNA samples were tested for quantity and quality using a Nanodrop 2000 Spectrophotometer (Thermo Scientific, USA). F_1_ individuals showing double or single flower were selected. The BSA extreme pools were divided based on the single-petaled pool with five petals and the double-petaled pool with 15 or more petals. A total of 64 plants were selected for the double flower pool, and 41 plants were selected for the single flower pool. An equal amount of DNA sample was collected from F_1_ population by phenotype. The pooled DNAs were sequenced on an Illumina Hiseq X10 PE150 (Genedenovo Biotechnology Co., Ltd, Guangzhou, China).

The raw reads were first filtered to obtain high-quality clean reads. The clean reads were aligned to the *P. mume* reference genome using Burrows-Wheeler Aligner [36, 38], and the results were sorted and tagged with duplicated sequences using Picard (Http://sourceforge.net/projects/picard/). Coverage statistics were performed using bedtools (v2.27.1) [38]. The processed alignment files were used for variant calling, which was performed on each sample using GATK’s Variant Filtration with appropriate parameter settings. SNPs and INDELs were annotated by ANNOVAR software [39]. The Δ(SNP/InDel-index) and the Euclidean distance calculation were used for mapping candidate regions associated with double flower phenotype [40, 41].

### Cloning of *PmAP2L*, genotyping, and phylogenetic analyses

The coding sequence (CDS) of *PmAP2L* was cloned from the buds of the single flower (named *PmAP2L-S*) and double flower (named *PmAP2L-D*) varieties of F_1_ population (primers used are listed in Table S15, see online supplementary material). Double-flowered varieties of *P. mume* included ‘Longyou’ (LY) and ‘Fentai Chuizhi’ (FTCZ), and the single-flowered variety included ‘Liuban’ (LB). The fragments of the 49 bp variant in *PmAP2L* were cloned from the double flower pool and single flower pool of the F_1_ population, as well as from randomly selected pools of 10 double and 10 single flower varieties. A CAPS marker was developed based on the 49-bp variant in *PmAP2L*, and genotyping was performed in the double and single flower pools of the F_1_ population. We applied MUSCLE to align protein sequences under default parameters. The phylogenetic tree was constructed by using the Maximum Likelihood method in iqtree tool, and 2000 bootstrap replicates were set to assess the reliability of the phylogenetic tree.

### RNA extraction and qRT-PCR

Total RNA was obtained from each sample using the RNAprep Pure Plant Plus Kit (DP441, TIANGEN), and first-strand cDNAs were synthesized from 1 μg of total RNA using the PrimeScript™ RT reagent Kit with gDNA Eraser (RR047, TaKaRa, Bejing, China). Gene-specific primers used in qRT-PCR were designed by the NCBI primer tool (Table S15, see online supplementary material). The qRT-PCR was performed as described previously for the reaction system and conditions using the SYBR Premix Ex Taq II kit (RR820, TaKaRa) on a PikoReal real-time PCR system (Thermo Fisher Scientific, Waltham, MA, USA) [36, 42]. The relative expression levels of genes were calculated using the 2 ^– ΔΔCt^ formula with *PmPP2A*, *NtActin*, and *RcTCTP* as the internal reference genes in *P. mume*, tobacco and rose, respectively.

### GUS staining analysis

*35S::PmAP2L-D-GUS* and *35S::PmAP2L-S-GUS* constructs were generated by inserting the CDS of *PmAP2L-D/S* into the pCAMBIA1304-*GUS* vector through *Nco* I restriction site (Table S15, see online supplementary material). *P. mume* microR172a precursor gene (*Pmu-pre-miR172a*) was inserted into the pCAMBIA1304-GUS vector according to a previous sequence synthesis [43]. GUS staining was executed as previously specified [37].

### Dual luciferase reporter assay

The precursor sequences of *Pmu-miR172a* and other ornamental plant *miR172* and STTM172 were recombined into pGreenII0029 62-SK, whereas the *miR172* target site fragments of *PmAP2L-D/S* and homologous *AP2L*s were inserted into the pGreenII 0800-35S-*LUC*. *STTM172* was designed and synthesized according to the mature sequence of miR172 [44]. For the transcriptional activity assay, the CDS of *PmAP2L-D/S*, *PmTPL*, and *PmHDA6/19* were PCR amplified from cDNA of *P. mume* and cloned into the pGreenII 0029 62-SK vector to construct effectors, and the promoter sequences of *PmAP3–1*, *PmAP3–2*, *PmAG*, and *PmSEP3*. The second intron sequence of *PmAG* was amplified from gDNA and inserted into the pGreenII 0800-*LUC* vector to construct the reporter (Table S15, see online supplementary material). The LUC reporter assay was executed as previously specified [31].

### Subcellular localization

*35S::PmAP2L-D-GFP* and *35S::PmAP2L-S-GFP* constructs were generated by inserting the CDS of *PmAP2L-D/S* into the pCAMBIA1300super vector through *Kpn* I restriction site (Table S15, see online supplementary material). Subcellular localization was performed according to a method described previously [37].

### Tobacco transformation

*35S::PmAP2L-D* and *35S::PmAP2L-S* constructs were generated by inserting the CDS of *PmAP2L-D/S* into the pCAMBIA1304 vector through *Nco* I and *Bst* EII restriction sites (Table S13, see online supplementary material). The constructed vectors were transformed through *Agrobacterium tumefaciens*-mediated transformation in tobacco leaves and positive lines were confirmed by qRT-PCR as described previously [42].

### Virus-induced gene silencing assay of rose

The CDS of *RC5G0530900* was PCR amplified from cDNA of *R. chinensis* ‘Old blush’. A gene-specific fragment of *RC5G0530900* (234 bp in length) was inserted into the *Kpn* I-*Sac* I site of pTRV2 vector to produce the pTRV2-*RC5G0530900* construct (Table S15, see online supplementary material). The rose VIGS experiments were executed as previously specified [45]. We collected newly emerged young leaves after silencing by VIGS for the qRT-PCR analysis.

### Transient gene expression in *P. mume* flower buds

Transiently overexpressing *PmAP2L-D/S* and *PmMIR172a* was performed using the *35S::PmAP2L-D/S* and *35S::Pmu-pre-172a* constructs that were used in tobacco transformation. For VIGS, a gene-specific fragment of *PmAP2L* (234 bp in length) and STTM172 was inserted into the *Kpn* I-*Sac* I site of pTRV2 vector to produce the pTRV2-*PmAP2L* and pTRV2-STTM172 constructs, respectively (Table S15, see online supplementary material). Transient transformation experiments were executed as previously specified with some modifications.

The constructs were then transformed into *A. tumefaciens* GV3101 cells and cultured in Luria-Bertani medium with 50 mg/L kanamycin and 25 mg/L rifampicin. The cells were resuspended in buffer (10 mM MgCl_2_, 10 mM 2-(N-Morpholino)-ethanesulfonic acid, pH = 5.8, 150 μM acetosyringone) until OD600 = 0.8, and then placed at room temperature in the dark for 4 h. 'Jiangmei' shoots were collected from natural conditions in mid-November 2022 and then cut into single-node shoots at room temperature conditions. The junction of the bud and branch were pierced with syringe needles. Subsequently, the shoots were soaked in the infiltration buffer and exposed to vacuum for 15 min. The infiltrated shoots were washed with distilled water and placed on water-soaked floral foam. The floral foams were placed in the dark for 24 h and then incubated at 25°C/23°C (light/dark). After 5 days of infection, the flower buds were collected for the qRT-PCR analysis.

### DAP-seq

The genomic DNA was extracted from the leaves of *P. mume* using the CTAB method to prepare the DNA library. The CDS of *PmAP2L-D* was inserted into the HaloTag expression vector to generate recombinants. A mixture of the Magne HaloTag Beads and Halo-*PmAP2L-D* was incubated with the DNA library. The DNA fragment bound to the *PmAP2L* was eluted from the beads and sequenced on the Illumina NovaSeq 6000 platform. The clean reads were aligned to the *P. mume* reference genome using BWA-MEM [46]. The sequenced data were identified for peaks using MACS [47]. We employed MEME-ChIP in order to identify the core motifs [48]. DAP-seq was executed as previously specified [49, 50].

### Yeast one-hybrid assay

The CDS of *PmAP2L-D/S* was inserted into the *Eco* R I site of the pGADT7 as prey. The promoter sequences of *PmAP3–1*, *PmAP3–2*, *PmAG*, and *PmSEP3*, the second intron sequence of *PmAG*, and its partial sequences (with AP2 binding sites predicted by PlantPAN 3.0), containing a triplication of AP2 binding site variants plus three nucleotides of the flanking sequence on both sides from *pPmAG* and *inPmAG*, were ligated in the *Sac* I-*Kpn* I sites of the pAbAi vector as baits (Table S15, see online supplementary material). A total of 19 recombinant plasmids were transformed into the Y1H Gold strain after being linearized by *Bst* BI, and their auto-activation was detected on the SD/-Ura/AbA medium. Subsequently, the Y1HGold strain without auto-activation was re-transformed with the pGADT7-*PmAP2L-D/S* vector or empty vector. The transformant cells were then spotted on the SD/−Leu + 800 ng/mL AbA medium and cultured for 72 h to identify DNA-protein interactions.

### Yeast two-hybrid assay

The CDSs of *PmTPL*, *PmAP2L-D/S*, and *PmHDA6/19* were inserted into the *Eco* R I site of the pGBKT7 as baits. The CDSs of *PmAP2L-D*, *PmAP2L-D-mEAR1* (mutation of the first EAR motif), *PmAP2L-D-mEAR2* (mutation of the second EAR motif), *PmAP2L-D-mEAR3* (mutation of all EAR motif), *PmAP2L-S*, *PmAP2L-S-mEAR1/2/3*, and *PmTPL* were inserted into the *Eco* R I site of the pGADT7 as preys (Table S15, see online supplementary material). Subsequently, the various preys and baits were co-transformed into the yeast strain Y2Hgold. The transformants were then cultured on different media (SD/−Trp/−Leu; SD/−Trp/−Leu/-His/−Ade + 3AT; SD/−Trp/−Leu/-His/−Ade + 3AT + X-α-Gal) and grown for 72 h to identify the interaction between two proteins.

### Yeast three-hybrid assay

The CDS of *PmTPL* was inserted into the *Not* I-*Bgl* II sites of the pBridge vector to generate the pBridge-PmTPL plasmid (Table S15, see online supplementary material). Subsequently, the CDSs of *PmAP2L-D/S* and *PmHDA6/19* were cloned into the *Sal* I site of the pBridge-PmTPL plasmid to produce the pBridge-PmTPL-PmAP2L-D/S and pBridge-PmTPL-PmHDA6/19 vector as baits (Table S15, see online supplementary material). The CDSs of *PmAP2L-D/S* and *PmHDA6/19* were inserted into the *Eco* R I site of the pGADT7 as preys (Table S15, see online supplementary material). The Yeast three-hybrid assay was executed as previously specified [51].

### Firefly luciferase complementation imaging assay

The CDSs of *PmAP2L-D*, *PmAP2L-D-mEAR1/2/3*, *PmAP2L-S*, *PmAP2L-S-mEAR1/2/3* were cloned into the *Kpn* I-*Sal* I sites of the pCAMBIA1300-*nLUC* (Table S15, see online supplementary material). The CDSs of *PmTPL* and *PmHDA6/19* were cloned into the *Kpn* I-*Sal* I sites of the pCAMBIA1300-cLUC (Table S15, see online supplementary material). Luciferase complementation imaging assay was executed as previously specified [31].

## Supplementary Material

Web_Material_uhad278

## Data Availability

The raw sequence data of BSA-seq is available in the National Genomics Data Center (NGDC) with the accession number PRJCA017066.The DAP-seq data have been deposited to the National Genomics Data Center (NGDC) with the accession number PRJCA017094.
