# Supplementary material for: A 49-bp deletion of PmAP2L results in a double flower phenotype in Prunus mume
Source: Hortic Res. 2023 Dec 19;11(2):uhad278. doi: 10.1093/hr/uhad278 (PMC10873580; doi:10.1093/hr/uhad278)
Supplement: Web_Material_uhad278 [file web_material_uhad278.zip › Supporting Information 20231203.pdf]

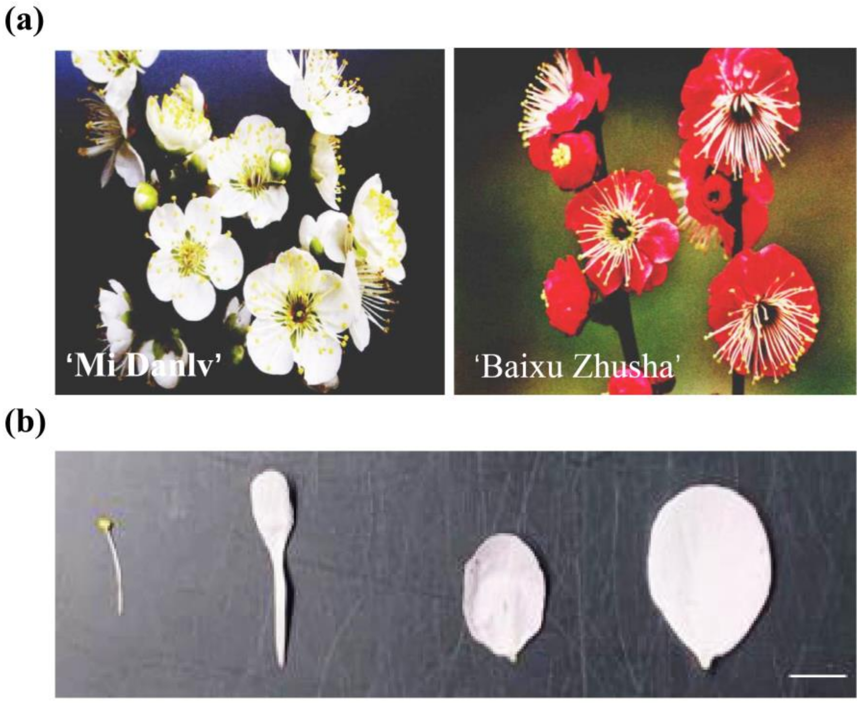

Supplementary Fig. S1 Flower morphology in *P. mume*. (a) Flower morphology 'Mi Danlv' and 'Baixu Zhusha'. (b) Variant petal morphology in the F_1 segregation population.

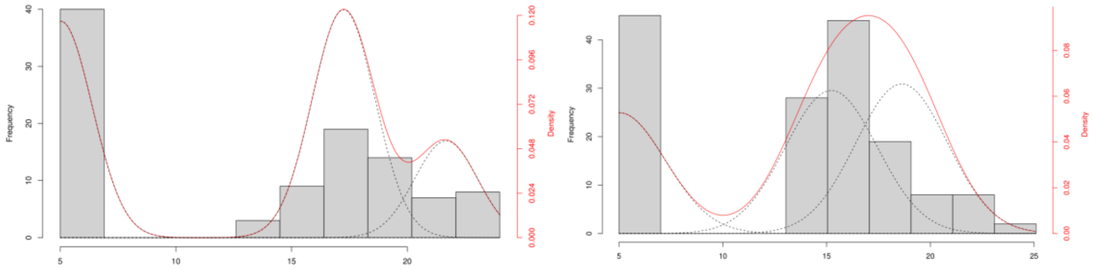

Supplementary Fig. S2 Histogram of the number of petals compared with the theoretical distribution curve of the optimal model. The left figure is the 2021 comparison chart, and the right figure is the 2022 comparison chart.

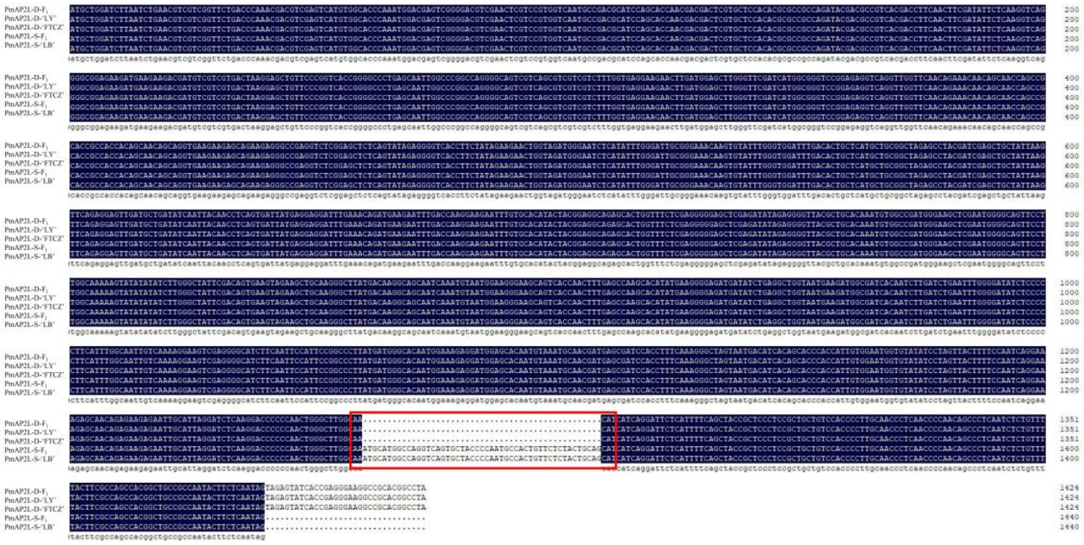

Supplementary Fig. S3 Sequence alignment of CDS sequence of *PmAP2L* in different flower type varieties of *P. mume*. *PmAP2L-D*-F_1 and *PmAP2L-S*-F_1 represent CDS sequence of *PmAP2L* in double flower and single flower of F_1 population, respectively. *PmAP2L-D*-'LY', *PmAP2L-D*-'FTCZ', and *PmAP2L-S*-'LB' represent CDS sequence of *PmAP2L* in 'LY', 'FTCZ', and 'LB', respectively. 'LY' and 'FTCZ' are double flower varieties. 'LB' is single flower variety.

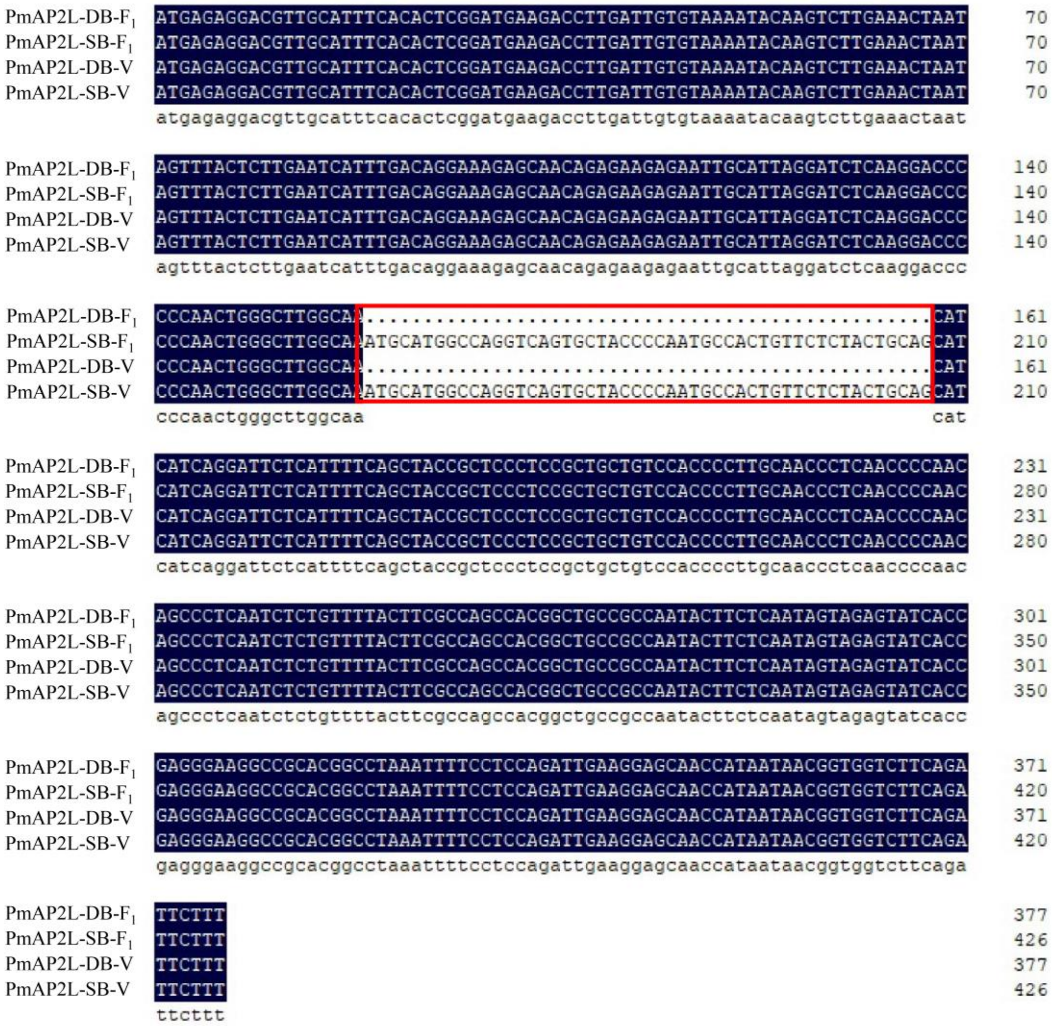

Supplementary Fig. S4 Sequence alignment of partial sequence of *PmAP2L* in gDNA. *PmAP2L-DB*-F_1 and *PmAP2L-SB*-F_1 represent partial sequence of *PmAP2L* in gDNA in double flower bulk and single flower bulk of F_1 population, respectively. *PmAP2L-DB*-V and *PmAP2L-SB*-V represent partial sequence of *PmAP2L* in gDNA in double flower bulk and single flower bulk of varieties, respectively.

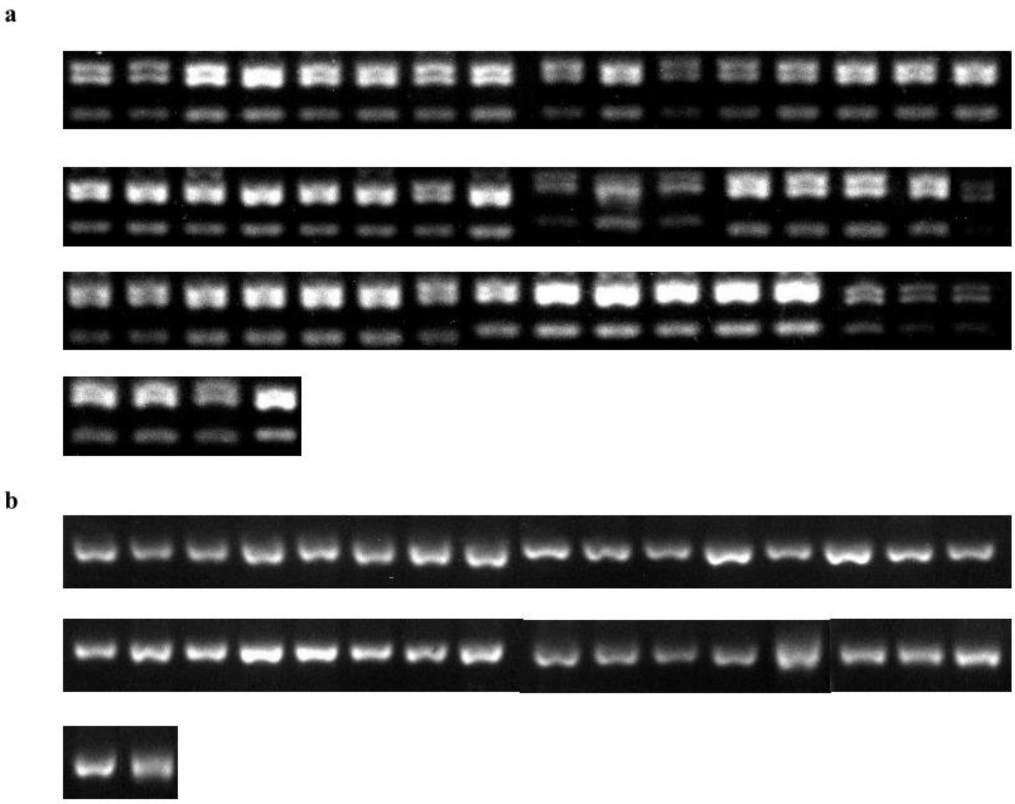

Supplementary Fig. S5 a cleaved amplified polymorphic sequence (CAPS) marker was developed based on the 49-bp deletion and validated in F_1 population. (a) Double flower individuals. (b) Single flower individuals.

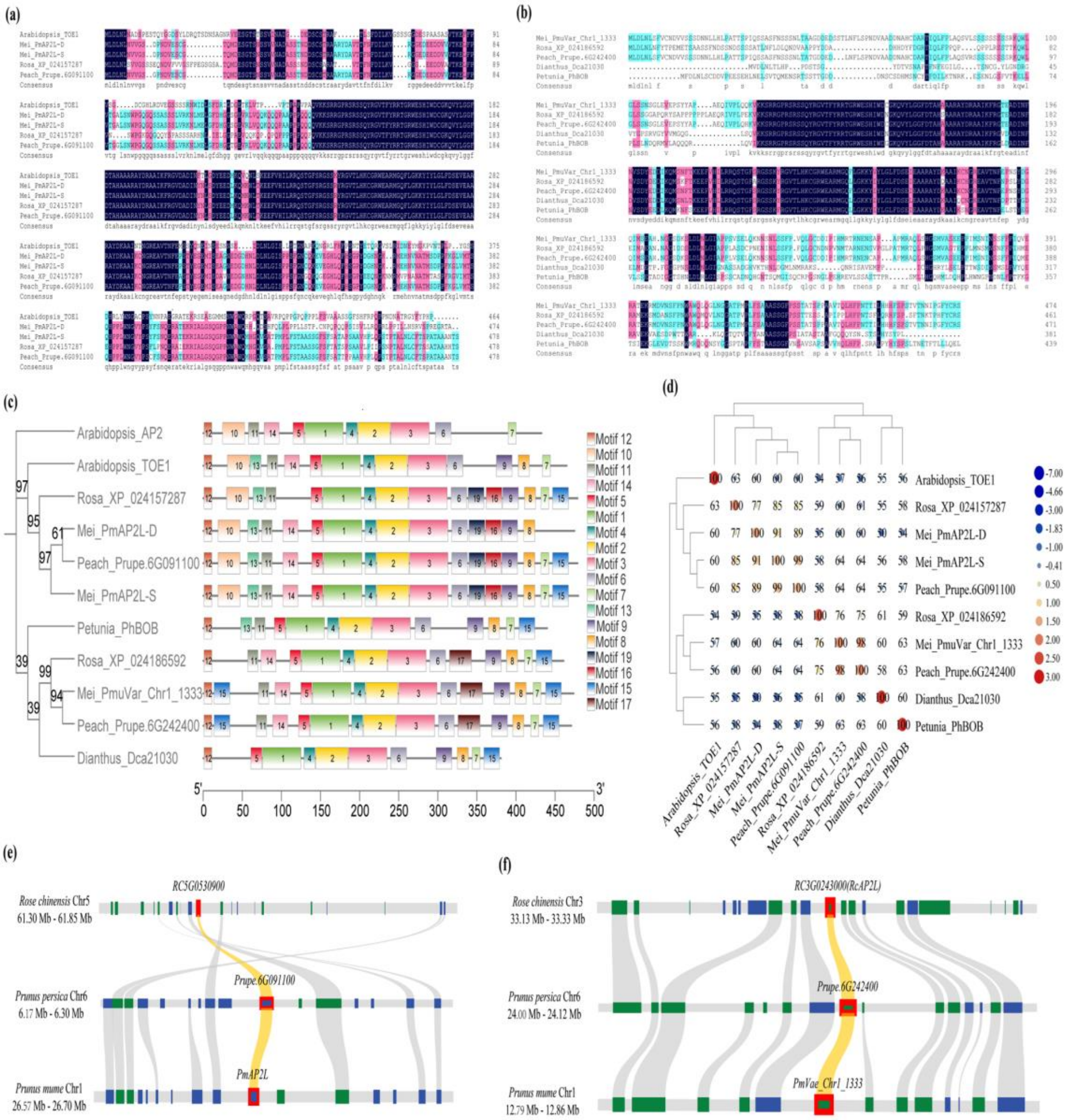

Supplementary Fig. S6 Sequence alignment, conserved domain distribution, similarities and microsynteny analysis of the euAP2 gene subfamily. (a) Alignment of putative amino acid sequences of PmAP2L-D/S protein and three other species. (b) Alignment of putative amino acid sequences of PmuVar_Chr1_1333 protein and four other species. (c) Conserved domain distribution of the euAP2 gene subfamily. (d) Similarities between members of the euAP2 gene subfamily. (e) Microsynteny analysis of *AP2L* locus in *Prunus mume*, *Rosa chinensis*, and *Prunus persica.* (f) Microsynteny analysis of *PETALOSA* TOE-type genes locus in *Prunus mume*, *Rosa chinensis*, and *Prunus persica.*

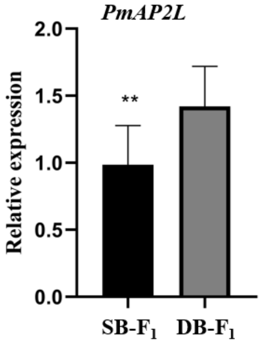

Supplementary Fig. S7 Gene expression analysis of *PmAP2L* in single flower (SB-F_1) and double flower bulk (DB-F_1) of F_1 population. Data are represented as the mean of biological triplicates ± SD (n=6). Different letters above the bars indicate a significant difference ($P < 0.05$, Student's t test).

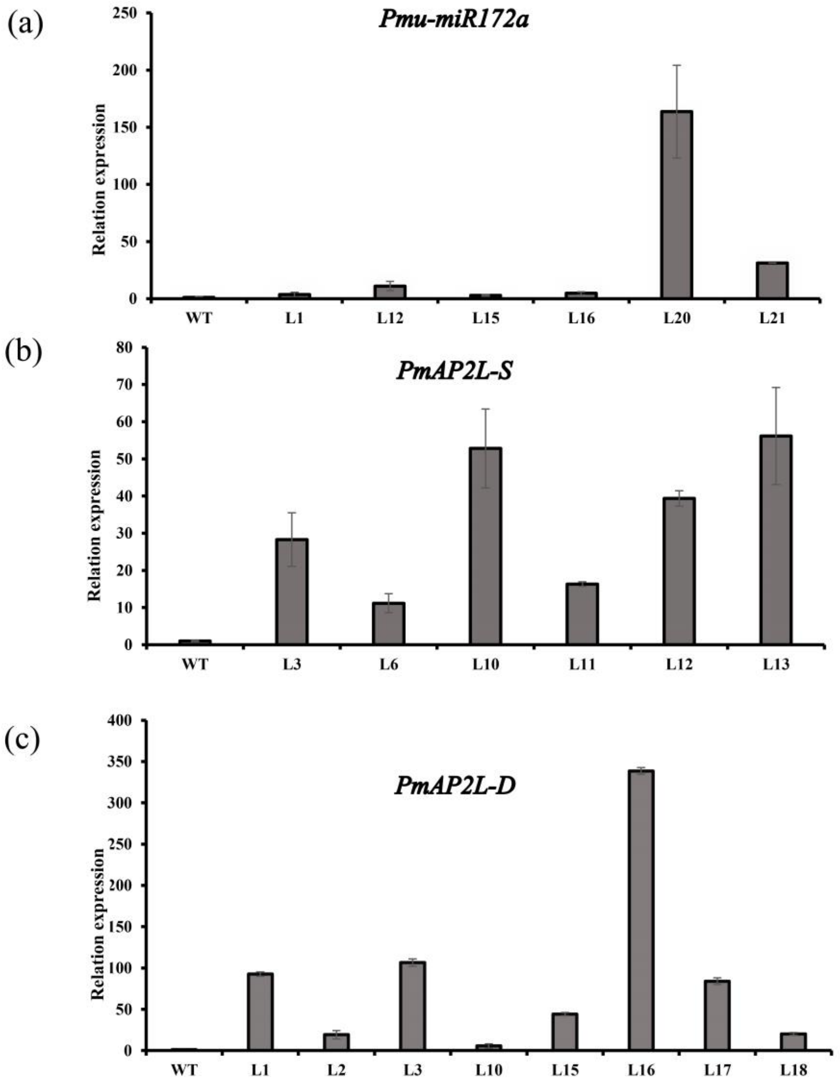

Supplementary Fig. S8 Expression of *miR172a*, *PmAP2L-S* and *PmAP2L-D* genes in transgenic tobacco lines. (a) miR172a expression detection in *35S::Pmu-pre-172a* transgenic tobacco, WT is wild-type

tobacco. (b) Detection of *PmAP2L-S* gene expression in *35S::PmAP2L-S* transgenic tobacco. (c) Expression assay of *PmAP2L-D* gene in *35S::PmAP2L-D* transgenic tobacco. The mean ± SD from three biological replicates (n =3) are shown.

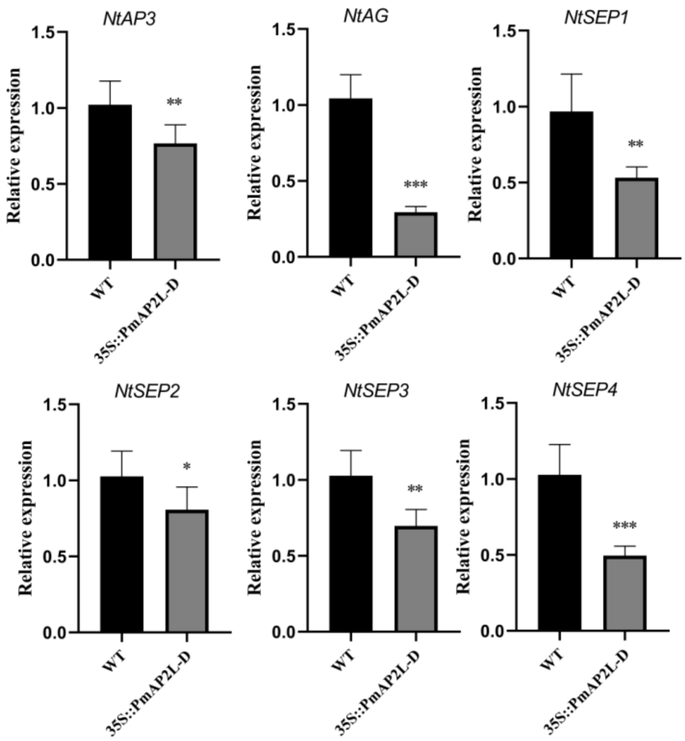

Supplementary Fig. S9 qRT-PCR analysis of endogenous genes related to flower development in transgenic tobacco. The mean ± SD from three biological replicates (n =3) are shown. Asterisks indicate statistically significant differences (two-sided Student's t test; **P* < 0.05, ***P* < 0.01 and ****P* < 0.001).

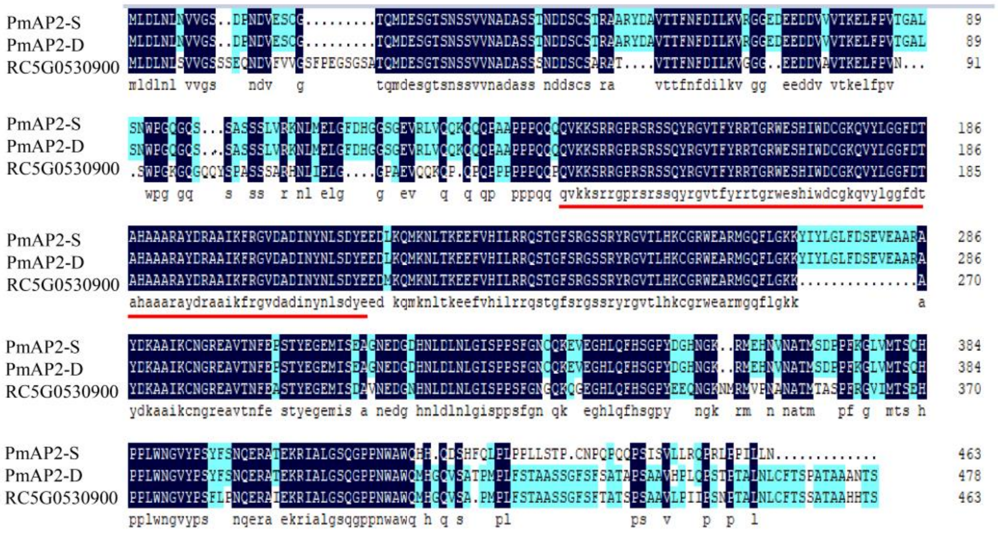

Supplementary Fig. S10 Alignment of putative amino acid sequences of PmAP2L-D/S and RC5G05309 proteins.

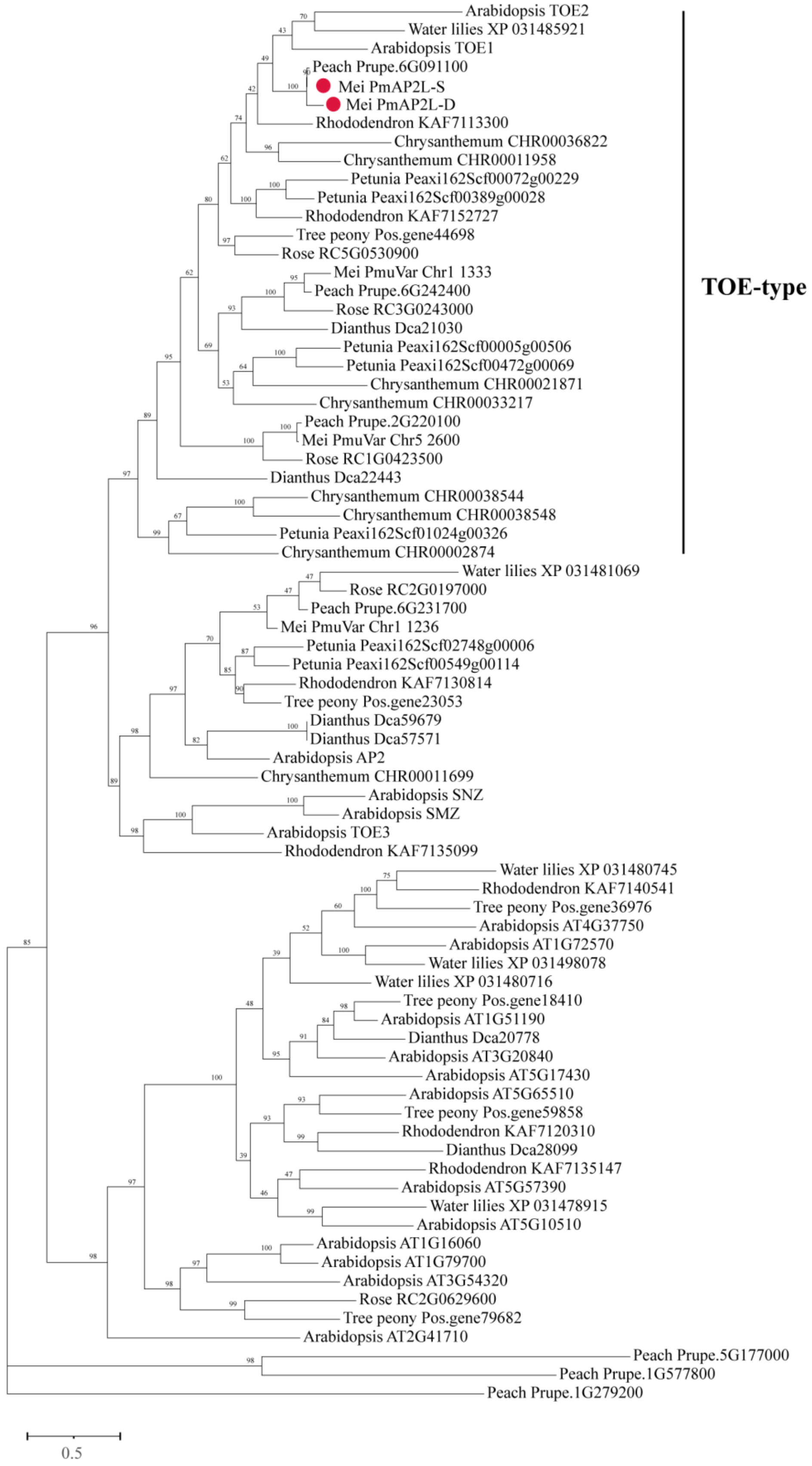

Supplementary Fig. S11 Phylogenetic tree analysis of miR172 targeted AP2s in plants. (rectangle)

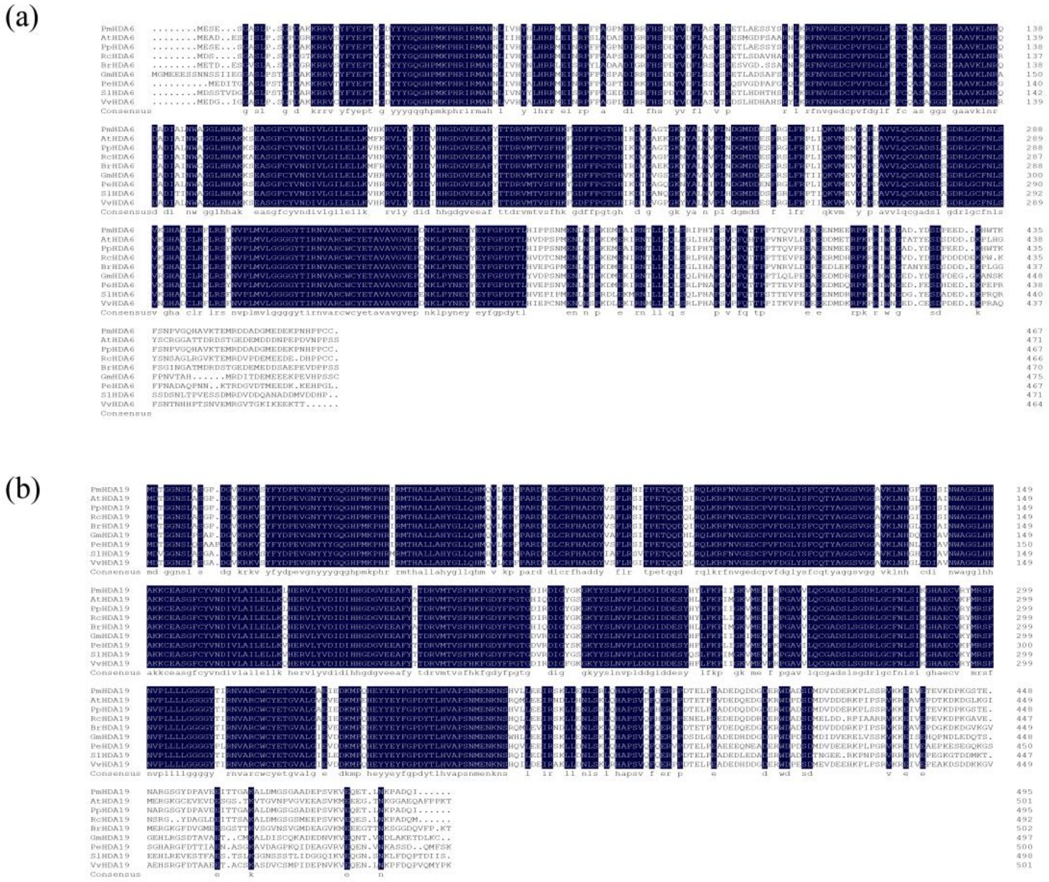

Supplementary Fig. S12 Sequence alignment of PmHDA6/19. (a) Alignment of putative amino acid sequences of PmHDA6 protein and eight other species. (b) Alignment of putative amino acid sequences of PmHDA19 protein and eight other species.

Supplementary Table S1 Descriptive statistics of petal number in the F_1 hybrid population

Trait	P1	P2	F_1 hybrid population			Skewness	Kurtosis	CV （%）
			Max	Min	Mean			
Petal number-2021	5	24.1	24	5	13.15	-0.17	-1.69	52.9
Petal number-2022	5	24.1	25.1	5	13.47	-0.5	-1.08	43

Supplementary Table S2 A kaike's Information Criterion (AIC) value under 11 genetic models for petal number in the F_1 hybrid population

Model code		Petal number-2021		Petal number-2022	
	Model implication	Log max-likelihood-value	AIC value	Log max-likelihood-value	AIC value
A-0	0MG	-335.347	674.6944	-488.532	981.0639
A-1	1MG-AD	-148.678	305.355	-224.846	457.6914
A-2	1MG-A	-301.986	609.9715	-430.323	866.6451
A-3	1MG-EAD	-152.323	312.645	-268.786	545.5716
A-4	1MG-NCD	-335.349	678.697	-488.532	985.0649
B-1	2MG-ADI	-214.739	449.4773	-333.117	686.2338
B-2	2MG-AD	-	-	-	-
B-3	2MG-A	-	-	-	-
B-4	2MG-EA	908.3479	-1810.7	902.217	-1798.43
B-5	2MG-CD	-276.447	560.8949	-415.139	838.2784
B-6	2MG-EAD	-276.446	558.8909	-415.138	836.2762

Supplementary Table S3 Test for fitness of the genetic models of petal number in the F_1 hybrid population.

Trait	Model code	U_1^2	$P(U_1^2)$	$U2^2$	$P(U2^2)$	$U3^2$	$P(U3^2)$	nW^2	$P(nW^2)$	Dn	P(Dn)
Petal number-2021	A-1	0.121	0.7279	0.036	0.8495	0.3467	0.556	0.4355	0.0601	0.2121	2.47E-04
	A-3	0.273	0.6013	0.153	0.6957	0.2109	0.6461	0.4615	0.0512	0.2121	2.47E-04
Petal number-2022	A-1	0.082	0.7747	0.057	0.8113	0.0236	0.878	0.3543	0.1005	0.1461	0.0028
	A-3	1.086	0.2974	1.4034	0.2362	0.494	0.4822	0.7709	0.0086	0.1461	0.0028

Note: The uniformity test consists of $U1^2$, $U2^2$ and $U3^2$; The Smirnov test and the Kolmogorov test are nW^2 and Dn, respectively; P represents the corresponding *P* value.

Supplementary Table S4 Genetic parameters of alternative models.

Trait	Model code	*m*	*da(d)*	*db*	*ha(h)*	*hb*	*i*	*jab*	*jba*	*l*	σmg^2	hmg^2
Petal number-2021	A-1	13.349	8.329	-	3.9004	-	-	-	-	-	46.5123	96.1415
	A-3	11.7944	6.7744	-	-	-	-	-	-	-	41.9843	86.782
Petal number-2022	A-1	11.8206	6.8006	-	3.4087	-	-	-	-	-	28.6728	85.4499
	A-3	10.9912	5.9713	-	-	-	-	-	-	-	28.1314	83.8366

Note: *m* represents the average square of the group; *da* and *db* represent additive effect of the first and second major genes, respectively; *ha* and *hb* represent dominant effect of the first and second major genes, respectively; *i*, *jab*, *jba* and *l* represent additive × additive effect, additive × dominance effect, dominance × additive effect, and dominance × dominance effect, respectively; σmg^2 and hmg^2 represent major gene variance and heritability, respectively.

Supplementary Table S5 Sample sequencing data statistics

Sample ID	Raw reads	Clean base （GB）	HQ Clean bsae （GB）	GC rate （%）	Q20 rate （%）	Q30 rate （%）
Double-Pool	112541216	16.88	16.59	39.68	96.70	91.75
Single-Pool	83122734	12.46	12.18	40.17	96.34	91.26

Supplementary Table S6 Sample alignment results.

Sample ID	Average depth （×）	Total mapped rate (%)	Clean mapped rate (%)	Cover 4× (%)	Cover 10× (%)
Double-Pool	71	97.86	80.87	94.90	92.48
Single-Pool	52	97.77	80.26	94.25	90.28

Supplementary Table S7 SNP information statistics in samples.

Sample ID	SNP number	TS	TV	TS/Tv	Het	Hom	Heterozygosity rate (%)
Double-Pool	1589316	1022450	566866	1.8037	1349858	239458	84.93
Single-Pool	1583269	1019167	564102	1.8067	1342757	240512	84.81

Supplementary Table S8 Statistics of IN/DEL information in samples.

Sample ID	In/Del number	Het	Hom	Heterozygosity rate (%)
Double-Pool	318316	169442	148874	53.23
Single-Pool	314603	168682	145921	53.62

Supplementary Table S9 Candidate intervals under the 99% confidence threshold for the SNP-index method.

Chromosome	Start	End	Candidate region（Mb）
Chr1	23190001	26570000	3.38
Chr1	26720001	27310000	0.59

Supplementary Table S10 Candidate intervals under the 99% confidence threshold for the ED method.

Chromosome	Start	End	Candidate region（Mb）
Chr1	24390001	27420000	3.03

Chr1	30180001	31170000	0.99

Supplementary Table S11 Candidate intervals under the 95% confidence threshold for the SNP-index method.

Chromosome	Start	End	Candidate region（Mb）
Chr1	820001	1360000	0.54
Chr1	21500001	31160000	9.66
Chr2	31990001	32680000	0.69
Chr2	39720001	40380000	0.66
Chr2	41330001	42230000	0.90
Chr2	42410001	45370000	2.96
Chr8	420001	1010000	0.59

Supplementary Table S12 Candidate intervals under the 95% confidence threshold for the ED method.

Chromosome	Start	End	Candidate region（Mb）
Chr1	560001	1470000	0.91
Chr1	21450001	31180000	9.73
Chr2	37970001	38510000	0.54
Chr2	39740001	40370000	0.63
Chr2	40550001	41090000	0.54
Chr2	43130001	45400000	2.27
Chr8	350001	1050000	0.70

Supplementary Table S13 Primers used in this study.

Purpose	Primer sequence (5′−3′)
**For qRT-PCR**	
PmAP1-RT-F	GGAGAAGGAGAAGGCCGC
PmAP1-RT-R	TGGAAGCGGCTGTGGAAG
PmAP2-RT-F	TGAAGAAGAGCCGGCGTG
PmAP2-RT-R	TGAGACTCCCATCGGCCA
PmTOE3-RT-F	TCGTCTCCAGCTCCGACA
PmTOE3-RT-R	CGGCAGTGAGGTTGGAGG
PmAP3-1-RT-F	CGCGACCAATAGGCAGGT
PmAP3-1-RT-R	TGGTGGAGGGGCTGATGT
PmAP3-2-RT-F	GCATTGGCAAATGGGGCG
PmAP3-2-RT-R	GCGAACTTCCTCCGTGGT
PmPI-RT-F	TGCAGCCCTTCAGTCACG
PmPI-RT-R	GCCTGAGCTCGACTTGCA

PmAG1-RT-F	GCAAAAGGCGCAATGGGT
PmAG1-RT-R	TCATAAAGTCGGCCGCGG
PmAG2-RT-F	TGGGCCAGAGCTGAATGC
PmAG2-RT-R	AGGTTGCTCCACCCTCCA
PmSEP1-RT-F	CGATGCTGAGGTTGCCCT
PmSEP1-RT-R	CGGGTCTGTTGGCTTCCA
PmSEP2-RT-F	TGGGGAGGGGAAGAGTGG
PmSEP2-RT-R	AGCAACCTCAGCATCGCA
PmSEP3-RT-F	GGGAGGGGGAGAGTGGAG
PmSEP3-RT-R	AGCCCGTTCCTTCGCTTC
PmSEP4-RT-F	AGGAATGTGACGCAGCCA
PmSEP4-RT-R	TTGAGCAGCCTGGTGGTG
PmAP2L-RT-F	CTGAGGCTGGTAATGAAGATGG
PmAP2L-RT-R	TAAGGGCCGGAATGGAATTG
PmPP2A-RT-F	AGGGTTCGGCTCGCAATAATAGA
PmPP2A-RT-R	AGCAGCAGCATCACGAATTGAGTAG
NtAG-RT-F	CAACAGTGTAAGGGCGACCA
NtAG-RT-R	AAACTGCTCAGTGCCTCTCC
NtSEP1-RT-F	AGAAATTGTGGCTTGAGGCT
NtSEP1-RT-R	CCCAATTTGCAAAATAGAGTTGC
NtSEP2-RT-F	ATCCACCAGGGAGAAGTTGTG
NtSEP2-RT-R	TGCAAAGTGGAGTTGCATTCT
NtSEP3-RT-F	AGCCAACTAAATCTGCAGTGG
NtSEP3-RT-R	CCTGCTCCTCCTACTGTTATTGG
NtSEP4-RT-F	TCTGTTCTGTGCGATGCTGA
NtSEP4-RT-R	CAGTAGCTGATTGGCTGGCT
NtAP1-2-RT-F	GCTTTGTGATGCTGAAGTTGC
NtAP1-2-RT-R	CTCCAGTTTTCCTGCACCGA
NtActin-RT-F	TGTGTTGGACTCTGGTGATG
NtActin-RT-R	CGCTCGGTAAGGATCTTCATC
miR172-RT-F	AGAATCTTGATGATGCTGCAT
RcTCTP-RT-F	GGGTGATGATGCAGCTTT
RcTCTP-RT-R	TTAGCACTTGACCTCCTTCA
RC5G0530900-RT-F	GTCGTCGGAACAAAACGACG
RC5G0530900-RT-R	TCCTCCACCGACCTTGAGAA
**For clone**	
PmAP2L-D-CDS-F	ATGCTGGATCTTAATCTGAACGTCG
PmAP2L-D-CDS-R	TTAGGCCGTGCGGCCTTC
PmAP2L-S-CDS-F	ATGCTGGATCTTAATCTGAACG
PmAP2L-S-CDS-R	CTATTGAGAAGTATTGGCGGC
pPmAP3-1-F	TTCAGTCTTTCAAACTTCTGAAGAGCG
pPmAP3-1-R	GGCATCACAGAGAACAGTGAGCTCAT
pPmAP3-2-F	GTCTCTGAATATACCATTTTAGTTGCCGG
pPmAP3-2-R	GCCTTCTTGAAAATCCCATTTCTTCTC

pPmAG-F	CTCAAGTTCACATCTACTTTCAATGCCT
pPmAG-R	TTAGAGAAGACTATGAGAGCAACCTCTGC
pPmSEP3-F	GGTCGATCACCATTAATTAGTAGGACAC
pPmSEP3-R	GGTCGATCACCATTAATTAGTAGGACAC
inPmAG-F	CACAACGAACCGTCAAGTCACC
inPmAG-R	AGCTTCGGAAACAGATCCGGTA
PmTPL-CDS-F	ATGTCTTCTCTCAGCAGGGAGCTT
PmTPL-CDS-R	TCATCTCTGTGCTTGATCTGAACCT
PmHDA6-CDS-F	ATGGAGTCGGAAAGCGGGG
PmHDA6-CDS-R	TCAGCAGCACGGAGGATGATTT
PmHDA19-CDS-F	ATGGACACCGGCGGCAACTC
PmHDA19-CDS-R	TTATATCTGATCAGCAGGCTTATTCAGAGT
RC5G0530900-CDS-F	ATGTTGGATCTTAATCTCAGCG
RC5G0530900-CDS-R	TTATTGAGACGTATGGTGGGC
**For CAPS**	
PmAP2L-CAPS-F	TTCATTTAAGGTATGAGAGGAC
PmAP2L-CAPS-R	CTATTGAGAAGTATTGGCGG
**For subcellular localization**	
1300-*PmAP2L-D*-F	AAATACTAGTGGATCCGGTACCATGCTGGATCTTAATCTGAACG
1300-*PmAP2L-D*-R	CCCTTGCTCACCAT GGTACCGGCCGTGCGGCCTTC
1300-*PmAP2L-S*-F	AAATACTAGTGGATCCGGTACCATGCTGGATCTTAATCTGAACG
1300-*PmAP2L-S*-R	CCCTTGCTCACCAT GGTACC TTGAGAAGTATTGGCGGC
**For tobacco transformation**	
1304-*PmAP2L-D*-F	GAACACGGGGGACTCTTGACATGCTGGATCTTAATCTGAACGTCG
1304-*PmAP2L-D*-R	ACGATCGGGGAAATTCGAGCTGGTCACCTTAGGCCGTGCGGCCTTC
1304-*PmAP2L-S*-F	GAACACGGGGGACTCTTGACATGCTGGATCTTAATCTGAACG
1304-*PmAP2L-S*-R	ACGATCGGGGAAATTCGAGCTGGTCACCCTATTGAGAAGTATTGGCGGC
**For TRV vector construction**	
pTRV2-*RC5G0530900*-F	CTCCATGGGGATCCGGTACCCAGGTGAAGAAGAGCAGAAGAGGG
pTRV2-*RC5G0530900*-R	GCCTCGAGACGCGTGAGCTCATCCTCCTCATAATCACTGAGG
**For GUS histochemical staining**	
1304-*PmAP2L-D-GUS*-F	GAACACGGGGGACTCTTGACATGCTGGATCTTAATCTGAACGTCG
1304-*PmAP2L-D-GUS*-R	TCTCCTTTACTAGTCAGATCTACCATGGAGGCCGTGCGGCCTTC
1304-*PmAP2L-S-GUS*-F	GAACACGGGGGACTCTTGACATGCTGGATCTTAATCTGAA

	CG
1304-*PmAP2L-S-GUS*-R	TCTCCTTTACTAGTCAGATCTACCATGGATTGAGAAGTATT GGCGGC
**For dual-luciferase assay**	
pGreenSK-*Pmu-pre-miR172a*-F	ACTAGTGGATCCCCCGGGTTGTTTGCGGGCGTAGCA
pGreenSK-*Pmu-pre-miR172a*-R	GAATTCCTGCAGCCCGGGTTTATTGCCGATGCAGCATCA
pGreenLUC-*35S-PmAP2L-D*-F	GAGAGGACAGCGGCCGCAATGACATCACAGCACCCACC
pGreenLUC-*35S-PmAP2L-D*-R	ACCGCGGTGGCGGCCGCTTGAGAAGTATTGGCGGCAGC
pGreenLUC-35S-PmAP2L-*S*-F	GAGAGGACAGCGGCCGCTTTTCCAATCAGGAAAGAGC
pGreenLUC-35S-PmAP2L-*S*-R	ACCGCGGTGGCGGCCGCTGAGAAGTATTGGCGGCAGC
pGreenSK-*PmAP2L-D*-F	ACTAGTGGATCCCCCGGGATGCTGGATCTTAATCTGAACGT CG
pGreenSK-*PmAP2L-D*-R	GAATTCCTGCAGCCCGGGTTAGGCCGTGCGGCCTTCC
pGreenSK-*PmAP2L-S*-F	ACTAGTGGATCCCCCGGGATGCTGGATCTTAATCTGAACGT CG
pGreenSK-*PmAP2L-S*-R	GAATTCCTGCAGCCCGGGCTATTGAGAAGTATTGGCGGCA GC
pGreenSK-*PmTPL*-F	ACTAGTGGATCCCCCGGGATGTCTTCTCTCAGCAGGGAGC
pGreenSK-*PmTPL*-R	GAATTCCTGCAGCCCGGGTCATCTCTGTGCTTGATCTGAA CC
pGreenSK-*PmHDA6*-F	ACTAGTGGATCCCCCGGGATGGAGTCGGAAAGCGGGG
pGreenSK-*PmHDA6*-R	GAATTCCTGCAGCCCGGGTCAGCAGCACGGAGGATG
pGreenSK-*PmHDA19*-F	ACTAGTGGATCCCCCGGGATGGACACCGGCGGCAAC
pGreenSK-*PmHDA19*-R	GAATTCCTGCAGCCCGGGTTATATCTGATCAGCAGGCTTAT TC
pGreenLUC-*pPmAP3-1*-F	GAATTCCTGCAGCCCGGGGGCTCTTGTTGCTGTGCCATG
pGreenLUC-*pPmAP3-1*-R	ACTAGTGGATCCCCCGGGCTCTCTCTCTCTGCTTTGCTTTC
pGreenLUC-*pPmAP3-2*-F	GAATTCCTGCAGCCCGGGTTATATAAAAAAAAAAATCATG TTT
pGreenLUC-*pPmAP3-2*-R	ACTAGTGGATCCCCCGGGGTTTCCTCTCTCAATTTCCTC
pGreenLUC-*pPmAG*-F	GAATTCCTGCAGCCCGGGAAAACAATTAGCTAGTGTGGGA A
pGreenLUC-*pPmAG*-R	ACTAGTGGATCCCCCGGGAGTTGCAAGCTGAATCAAGCAC
pGreenLUC-*pPmSEP3*-F	GAATTCCTGCAGCCCGGGAGCATGAGCTAAGGTAACTAAA ATG
pGreenLUC-*pPmSEP3*-R	ACTAGTGGATCCCCCGGGCTCTCTCTCTCTCCCCAAAAC
pGreenLUC-*inPmAG*-F	GAATTCCTGCAGCCCGGGGTATTGCTTTTAAGTTCCTCTTA A
pGreenLUC-*inPmAG*-R	ACTAGTGGATCCCCCGGGACTACTAGTTACCACAAAAGAA TGG
**For luciferase complementation imaging assays**	
cLUC-*PmTPL*-F	ACGCGTCCCGGGGCGGTACCATGTCTTCTCTCAGCAGGGA GC
cLUC-*PmTPL*-R	AGCTCTGCAGGTCGACTCATCTCTGTGCTTGATCTGAACC

cLUC-*PmHDA6*-F	ACGCGTCCCGGGGCGGTACCATGGAGTCGGAAAGCGGGG
cLUC-*PmHDA6*-R	AGCTCTGCAGGTCGACTCAGCAGCACGGAGGATG
cLUC-*PmHDA19*-F	ACGCGTCCCGGGGCGGTACCATGGACACCGGCGGCAAC
cLUC-*PmHDA19*-R	AGCTCTGCAGGTCGACTTATATCTGATCAGCAGGCTTATTC
nLUC-*PmAP2L-D*-F	CGGGGGACGAGCTCGGTACCATGCTGGATCTTAATCTGAACGTCG
nLUC-*PmAP2L-D*-R	CGTATGGGTAGTCGACGGCCGTGCGGCCTTCCCT
nLUC-*PmAP2L-D-mEAR1*-F	CGGGGGACGAGCTCGGTACCATGGCGGATGCTAATGCGAACGTCGTCGGTTCTGAC
nLUC-*PmAP2L-D-mEAR1*-R	CGTATGGGTAGTCGACGGCCGTGCGGCCTTCCCT
nLUC-*PmAP2L-D-mEAR2*-F	CGGGGGACGAGCTCGGTACCATGCTGGATCTTAATCTGAACGTCG
nLUC-*PmAP2L-D-mEAR2*-R	CGTATGGGTAGTCGACGGCCGTGCGGCCTTCCCT
nLUC-*PmAP2L-D-mEAR3*-F	CGGGGGACGAGCTCGGTACCATGGCGGATGCTAATGCGAACGTCGTCGGTTCTGAC
nLUC-*PmAP2L-D-mEAR3*-R	CGTATGGGTAGTCGACGGCCGTGCGGCCTTCCCT
nLUC-*PmAP2L-S*-F	CGGGGGACGAGCTCGGTACCATGCTGGATCTTAATCTGAACGTCG
nLUC-*PmAP2L-S*-R	CGTATGGGTAGTCGACTTGAGAAGTATTGGCGGCAGC
nLUC-*PmAP2L-S-mEAR1*-F	CGGGGGACGAGCTCGGTACCATGGCGGATGCTAATGCGAACGTCGTCGGTTCTGAC
nLUC-*PmAP2L-S-mEAR*1-R	CGTATGGGTAGTCGACTTGAGAAGTATTGGCGGCAGC
nLUC-*PmAP2L-S-mEAR2*-F	CGGGGGACGAGCTCGGTACCATGCTGGATCTTAATCTGAACGTCG
nLUC-*PmAP2L-S-mEAR2*-R	CGTATGGGTAGTCGACTTGAGAAGTATTGGCGGCAGC
nLUC-*PmAP2L-S-mEAR3*-F	CGGGGGACGAGCTCGGTACCATGGCGGATGCTAATGCGAACGTCGTCGGTTCTGAC
nLUC-*PmAP2L-S-mEAR3*-R	CGTATGGGTAGTCGACTTGAGAAGTATTGGCGGCAGC
**For Y1H**	
pABAi-*pPmAP3-1*-F	AAAAGCTTGAATTCGAGCTCGGCTCTTGTTGCTGTGCCATG
pABAi-*pPmAP3-1*-R	TCGACAGATCCCCGGGTACCCTCTCTCTCTCTGCTTTGCTTTC
pABAi-*pPmAP3*-2-F	AAAAGCTTGAATTCGAGCTCTTATATAAAAAAAAAAATCATGTTT
pABAi-*pPmAP3*-2-R	TCGACAGATCCCCGGGTACCGTTTCCTCTCTCAATTTCCTC
pABAi-*pPmAG*-3-F	AAAAGCTTGAATTCGAGCTCGGCATAACAAACCAAATAGAACCCC
pABAi-*pPmAG*-3-R	TCGACAGATCCCCGGGTACCGATTCACTGCCGTAATTCAAAAGCC
pABAi-*pPmAG*-4-F	AAAAGCTTGAATTCGAGCTCAGAAAGTACCTTCTCTCTCTC
pABAi-*pPmAG*-4-R	TCGACAGATCCCCGGGTACCTTTAATTTTAAAGTATGTATTGGAG

pABAi-*pPmAG*-5-F	AAAAGCTTGAATTCGAGCTCAAAACAATTAGCTAGTGTGGGAA
pABAi-*pPmAG*-5-R	TCGACAGATCCCCGGGTACCAGTTGCAAGCTGAATCAAGCAC
pABAi-*pPmSEP3-1*-F	AAAAGCTTGAATTCGAGCTCCTTAGGACACACTTGTATTTG
pABAi-*pPmSEP3-1*-R	TCGACAGATCCCCGGGTACCATTATTTTAAGAAATAAAACATGTT
pABAi-*pPmSEP3-2*-F	AAAAGCTTGAATTCGAGCTCAGTTAAAATGGCAAATAAGGTGAA
pABAi-*pPmSEP3-2*-R	TCGACAGATCCCCGGGTACCTGCAAAGGTCCCAAGGGA
pABAi-*pPmSEP3-3*-F	AAAAGCTTGAATTCGAGCTCAGCGTCCCTTGGGACCTTTG
pABAi-*pPmSEP3-3*-R	TCGACAGATCCCCGGGTACCGATGTGTGGATCCGACGGTG
pABAi-*pPmSEP3-4*-F	AAAAGCTTGAATTCGAGCTCGCTTTTCCCCTATTGGTCTAAGTC
pABAi-*pPmSEP3-4*-R	TCGACAGATCCCCGGGTACCGGTCCTCTGGTTTTGCTTAGGG
pABAi-*pPmSEP3-5*-F	AAAAGCTTGAATTCGAGCTCAGCATGAGCTAAGGTAACTAAAATG
pABAi-*pPmSEP3-5*-R	TCGACAGATCCCCGGGTACCCTCTCTCTCTCTCCCCAAAAC
pABAi-*inPmAG*-2-F	AAAAGCTTGAATTCGAGCTCCAAAGGCATAGGATAAAGTGAAATC
pABAi-*inPmAG*-2-R	TCGACAGATCCCCGGGTACCTGAAATTAATTGTCGAGGGGG
pABAi-*inPmAG*-3-F	AAAAGCTTGAATTCGAGCTCGTATTGCTTTTAAGTTCCTCTTAA
pABAi-*inPmAG*-3-R	TCGACAGATCCCCGGGTACCACTACTAGTTACCACAAAAGAATGG
**For Y2H**	
pGBKT7-*PmTPL*-F	CATGGAGGCCGAATTCATGTCTTCTCTCAGCAGGGAGC
pGBKT7-*PmTPL*-R	GGATCCCCGGGAATTCTCATCTCTGTGCTTGATCTGAACC
pGBKT7-*PmAP2L-D*-F	CATGGAGGCCGAATTCATGCTGGATCTTAATCTGAACGTCG
pGBKT7-*PmAP2L-D*-R	GGATCCCCGGGAATTCTTAGGCCGTGCGGCCTTCC
pGBKT7-*PmAP2L-S*-F	CATGGAGGCCGAATTCATGCTGGATCTTAATCTGAACGTCG
pGBKT7-*PmAP2L-S*-R	GGATCCCCGGGAATTCCTATTGAGAAGTATTGGCGGCAGC
pGBKT7-*PmHDA6*-F	CATGGAGGCCGAATTCATGGAGTCGGAAAGCGGGG
pGBKT7-*PmHDA6*-R	GGATCCCCGGGAATTCTCAGCAGCACGGAGGATG
pGBKT7-*PmHDA19*-F	CATGGAGGCCGAATTCATGTCTTCTCTCAGCAGGGAGC
pGBKT7-*PmHDA19*-R	GGATCCCCGGGAATTCTCATCTCTGTGCTTGATCTGAACC
pGADT7-*PmAP2L-D*-F	GGAGGCCAGTGAATTCATGCTGGATCTTAATCTGAACGTCG
pGADT7-*PmAP2L-D*-R	CACCCGGGTGGAATTCTTAGGCCGTGCGGCCTTCC
pGADT7-*PmAP2L-D-mEAR1*-F	GGAGGCCAGTGAATTCATGGCGGATGCTAATGCGAACGTC

	GTCGGTTCTGAC
pGADT7-*PmAP2L-D-mEAR1*-R	CACCCGGGTGGAATTCTTAGGCCGTGCGGCCTTC
pGADT7-*PmAP2L-D-mEAR*3-F	GGAGGCCAGTGAATTCATGGCGGATGCTAATGCGAACGTCGTCGGTTCTGAC
pGADT7-*PmAP2L-D-mEAR3*-R	CACCCGGGTGGAATTCTTAGGCCGTGCGGCCTTC
pGADT7-*PmAP2L-S*-F	GGAGGCCAGTGAATTCATGCTGGATCTTAATCTGAACGTCG
pGADT7-*PmAP2L-S*-R	CACCCGGGTGGAATTCCTATTGAGAAGTATTGGCGGCAGC
pGADT7-*PmAP2L-S-mEAR1*-F	GGAGGCCAGTGAATTCATGGCGGATGCTAATGCGAACGTCGTCGGTTCTGAC
pGADT7-*PmAP2L-S-mEAR1*-R	CACCCGGGTGGAATTCCTATTGAGAAGTATTGGCGGCAGC
pGADT7-*PmAP2L-S-mEAR3*-F	GGAGGCCAGTGAATTCATGGCGGATGCTAATGCGAACGTCGTCGGTTCTGAC
pGADT7-*PmAP2L-S-mEAR3*-R	CACCCGGGTGGAATTCCTATTGAGAAGTATTGGCGGCAGC
**For Y3H**	
pBridge-*PmTPL*-F	AGAAAGGTGGCGGCCGCATGTCTTCTCTCAGCAGGGAGC
pBridge-*PmTPL*-R	ATCAGCCCGAAGATCTTCATCTCTGTGCTTGATCTGAACC
pBridge-*PmTPL-PmAP2L-D*-F	CCGGGGATCCGTCGACATGCTGGATCTTAATCTGAACGTCG
pBridge-*PmTPL-PmAP2L-D*-R	TTGGCTGCAGGTCGACTTAGGCCGTGCGGCCTTCC
pBridge-*PmTPL-PmAP2L-S*-F	CCGGGGATCCGTCGACATGCTGGATCTTAATCTGAACGTCG
pBridge-*PmTPL-PmAP2L-S*-R	TTGGCTGCAGGTCGACCTATTGAGAAGTATTGGCGGCAGC
pBridge-*PmTPL-PmHDA6*-F	CCGGGGATCCGTCGACATGGAGTCGGAAAGCGGGG
pBridge-*PmTPL-PmHDA6*-R	TTGGCTGCAGGTCGACTCAGCAGCACGGAGGATG
pBridge-*PmTPL-PmHDA19*-F	CCGGGGATCCGTCGACATGGACACCGGCGGCAAC
pBridge-*PmTPL-PmHDA19*-R	TTGGCTGCAGGTCGACTTATATCTGATCAGCAGGCTTATTC

Supplementary Table S14 Genomic information and miRNA target sites of the euAP2 gene subfamily of *P. mume*.

Gene ID	Genome ID	Location	miRNA172 target sites
PmAP2L-D	PmuVar_Chr1_3490	Chr1:26631633:26635047	Yes
PmAP2L-S	PmuVar_Chr1_3490	Chr1:26631633:26635047	Yes
PmuVar_Chr1_1236	PmuVar_Chr1_1236	Chr1:12238660:12241371	Yes
PmuVar_Chr1_1333	PmuVar_Chr1_1333	Chr1:12828956:12831811	Yes
PmuVar_Chr5_2600	PmuVar_Chr5_2600	Chr5:24766452:24769119	Yes

Supplementary Table S15 Similarities between members of the euAP2 subfamily of *P. mume* and *Arabidopsis thaliana*.

	PmAP2	PmAP2	PmuVar	PmuVar	PmuVar	At	At	At	At

	L-D	L-S	_Chr1_1236	_Chr1_1333	_Chr5_2600	TOE1	TOE2	TOE3	AP2
PmAP2L-D	100%								
PmAP2L-S	91%	100%							
PmuVar_Chr1_1236	49%	51%	100%						
PmuVar_Chr1_1333	60%	64%	50%	100%					
PmuVar_Chr5_2600	54%	58%	44%	54%	100%				
AtTOE1	60%	60%	52%	57%	52%	100%			
AtTOE2	50%	53%	44%	49%	50%	53%	100%		
AtTOE3	41%	45%	47%	49%	43%	52%	35%	100%	
AtAP2	50%	52%	58%	51%	48%	53%	43%	59%	100%

Supplementary Table S16 Accession numbers of the genes used in this study.

Species	Gene name	Accession number
Prunus mume	*PmAP2L*	PmuVar_Chr1_3490
Prunus mume	*PmAP2*	PmuVar_Chr1_1236
Prunus mume	*PmTOE3*	PmuVar_Chr1_1333
Prunus mume	*PmAP1*	PmuVar_Chr2_2758
Prunus mume	*PmFLU1*	PmuVar_Chr4_2568
Prunus mume	*PmAP3-1*	PmuVar_Chr2_2006
Prunus mume	*PmAP3-2*	PmuVar_Chr8_1221
Prunus mume	*PmPI*	PmuVar_Chr2_0915
Prunus mume	*PmAG-1*	PmuVar_Chr3_0748
Prunus mume	*PmAG-2-*	PmuVar_Chr2_0369
Prunus mume	*PmSEP1*	PmuVar_Chr7_1308
Prunus mume	*PmSEP2*	PmuVar_Chr4_2566
Prunus mume	*PmSEP3*	PmuVar_Chr2_3439
Prunus mume	*PmSEP4*	PmuVar_Chr2_2759
Prunus mume	*PmTPL*	PmuVar_Chr4_0936
Prunus mume	*PmTPR1*	PmuVar_Chr2_0974
Prunus mume	*PmTPR2a*	PmuVar_Chr2_0971
Prunus mume	*PmTPR2b*	PmuVar_Chr2_4327
Prunus mume	*PmTPR2c*	PmuVar_Chr2_0387
Prunus mume	*PmTPR3a*	PmuVar_Chr1_2115
Prunus mume	*PmTPR3b*	PmuVar_Chr2_4839

Prunus mume	*PmTPR3c*	PmuVar_Chr5_3557
Prunus mume	*PmTPR4a*	PmuVar_Chr4_0977
Prunus mume	*PmTPR4b*	PmuVar_Chr8_0401
Prunus mume	*PmHDA2*	PmuVar_Chr2_1609
Prunus mume	*PmHDA5*	PmuVar_Chr7_1268
Prunus mume	*PmHDA6*	PmuVar_Chr7_0891
Prunus mume	*PmHDA8a*	PmuVar_Chr1_0128
Prunus mume	*PmHDA8b*	PmuVar_Chr5_0272
Prunus mume	*PmHDA9*	PmuVar_Chr1_3880
Prunus mume	*PmHDA14*	PmuVar_Chr3_3270
Prunus mume	*PmHDA15*	PmuVar_Chr7_1390
Prunus mume	*PmHDA19*	PmuVar_Chr6_1547
Arabidopsis thaliana	*AtTPL*	AT1G15750
Arabidopsis thaliana	*AtTPR1*	AT1G80490
Arabidopsis thaliana	*AtTPR2*	AT3G16830
Arabidopsis thaliana	*AtTPR3*	AT5G27030
Arabidopsis thaliana	*AtTPR4*	AT3G15880
Arabidopsis thaliana	*AtHDA2*	AT5G26040
Arabidopsis thaliana	*AtHDA5*	AT5G61060
Arabidopsis thaliana	*AtHDA6*	AT5G63110
Arabidopsis thaliana	*AtHDA7*	AT5G35600
Arabidopsis thaliana	*AtHDA8*	AT1G08460
Arabidopsis thaliana	*AtHDA9*	AT3G44680
Arabidopsis thaliana	*AtHDA14*	AT4G33470
Arabidopsis thaliana	*AtHDA15*	AT3G18520
Arabidopsis thaliana	*AtHDA17*	AT3G44490
Arabidopsis thaliana	*AtHDA18*	AT5G61070
Arabidopsis thaliana	*AtHDA19*	AT4G38130
Prunus persica	*PpHDA19*	XP_007200978
Prunus persica	*PpHDA6*	XP_007209104
Rosa chinensis	*RcHDA19*	XP_024163177
Rosa chinensis	*RcHDA6*	XP_024169961
Brassica rapa	*BrHDA19*	XP_009124989
Brassica rapa	*BrHDA6*	XP_009150370
Glycine max	*GmHDA19*	XP_003526730
Glycine max	*GmHDA6*	XP_003525556
Populus euphratica	*PeHDA19*	XP_011040853
Populus euphratica	*PeHDA6*	XP_011046214
Solanum lycopersicum	*SlHDA19*	XP_004247825
Solanum lycopersicum	*SlHDA6*	NP_001352081
Vitis vinifera	*VvHDA19*	RVW48338
Vitis vinifera	*VvHDA6*	XP_010663108
